# ROS–Responsive Ferrocenyl Amphiphilic PAMAM Dendrimers for On–Demand Delivery of siRNA Therapeutics to Cancer Cells

**DOI:** 10.3390/pharmaceutics16070936

**Published:** 2024-07-13

**Authors:** Peng Chen, Zhihui Wang, Xinmo Wang, Junni Gong, Ju Sheng, Yufei Pan, Dandan Zhu, Xiaoxuan Liu

**Affiliations:** Center of Advanced Pharmaceuticals and Biomaterials, State Key Laboratory of Natural Medicines, China Pharmaceutical University, Nanjing 211198, China; 1731070152@stu.cpu.edu.cn (P.C.); 3221071566@stu.cpu.edu.cn (Z.W.); xinmo7890@outlook.com (X.W.); 3221071592@stu.cpu.edu.cn (J.G.); shengju@stu.cpu.edu.cn (J.S.); 3222071603@stu.cpu.edu.cn (Y.P.)

**Keywords:** amphiphilic dendrimer, ROS responsive, siRNA therapeutics, siRNA delivery

## Abstract

Small interfering RNA (siRNA) therapeutics, characterized by high specificity, potency, and durability, hold great promise in the treatment of cancer and other diseases. However, the clinic implementation of siRNA therapeutics critically depends on the safe and on–demand delivery of siRNA to the target cells. Here, we reported a family of ferrocenyl amphiphilic dendrimers (**Fc-AmDs**) for on–demand delivery of siRNA in response to the high ROS content in cancer cells. These dendrimers bear ROS–sensitive ferrocene moieties in the hydrophobic components and positively chargeable poly(amidoamine) dendrons as the hydrophilic entities, possessing favorable safety profiles and ROS responsive properties. One of these ferrocenyl amphiphilic dendrimers, **Fc-C_8_-AmD 8A**, outperforms in siRNA delivery, benefiting from its optimal balance of hydrophobicity and hydrophilicity. Its ROS feature facilitates specific and efficient disassembly of its complex with siRNA in ROS–rich cancer cells for effective siRNA delivery and gene silencing. Moreover, **Fc-C_8_-AmD 8A** also integrates the features and beneficial properties of both lipid and dendrimer vectors. Therefore, it represents a novel on–demand delivery system for cancer cell–specific siRNA delivery. This work opens new perspectives for designing self–assembly nanosystems for on–demand drug delivery.

## 1. Introduction

RNA interference (RNAi), triggered by small interfering RNA (siRNA), is a sequence-specific post–transcriptional gene silencing process that provides a powerful approach to interfere with the function of any disease–associated protein in a highly selective manner [1,2]. Therefore, siRNAs have been emerging as promising therapeutic reagents for the treatment of inherited and acquired diseases [2]. So far, six siRNA drugs have been approved for marketing worldwide [3]. A growing number of clinical trials of RNAi–based solid tumors treatment further demonstrate the potential of siRNA therapeutics in treating cancer patients by specifically turning off gene expression that promotes cancer cell survival, migration, and tumorigenicity [4]. However, the therapeutic outcomes extremely rely on the safe and efficient delivery of siRNA therapeutics in target cells, as they are susceptible to nuclease degradation and renal clearance [5]. Moreover, siRNA molecules are hydrophilic biomacromolecules with strongly negative charge, which prevents them from spontaneously crossing cell membranes [6,7]. During the past decades, tremendous efforts have been dedicated to exploit delivery vehicles for siRNA therapeutics [5,6,7]. However, there is still an unmet need for the specific delivery of siRNA therapeutics to disease–related cells [7,8]. Given this, the development of on–demand delivery vehicles capable of transporting siRNA specifically to cancer cells is highly desirable and also of paramount importance for RNAi–based cancer therapy.

Lipids and polymers are the two most advanced vectors for siRNA therapeutics in clinical and preclinical studies [9]. For instance, the first siRNA drug approved by FDA, Onpattro [10], is a lipid nanoparticle formulation, while the first clinic trial of siRNA therapeutics is a polymer–based formulation [11]. Dendrimers, a special type of polymers, are emerging as promising delivery vehicles for siRNA therapeutics by virtue of their well-defined molecular structure and unique multivalent cooperativity [12,13]. In particular, amphiphilic dendrimers, a kind of lipid/dendrimer hybrid with judiciously designed hydrophilic and hydrophobic components, exhibit excellent performance on siRNA delivery thanks to the combined advantageous of lipid and polymer vectors as well as unique characteristics of dendrimer vectors [14,15]. We and others have recently established several types of amphiphilic dendrimers, including amphiphilic poly(amidoamine) (PAMAM) dendrimers [16,17], amphiphilic peptide dendrimers [18], amphiphilic poly(aminoester) dendrimers [19], and amphiphilic polyglycerol dendrimers [20], all of which possess the excellent capability of delivering siRNA therapeutics for the treatment of different diseases. Therefore, amphiphilic dendrimers are able to be a promising platform for elaborating on–demand vectors for cancer cell–specific siRNA delivery.

The tumor microenvironment has a variety of atypical hallmarks, such as hypoxia, abnormal redox environment, low pH, upregulated secretion of specific enzymes, overexpression of inflammatory meditators, etc. [21]. Notably, the hypoxia environment within the tumor tissues leads to the continuous production of reactive oxygen species (ROS) within cancer cells [22,23]. Thus, high levels of ROS are one of the intrinsic characteristics specifically associated with cancer cells [23,24]. Harnessing this factor to exploit on–demand vectors that are capable of ROS–triggered siRNA release in cancer cells constitute a promising strategy to achieve cancer cell–specific siRNA delivery for RNAi–based cancer therapy.

**Scheme 1:**
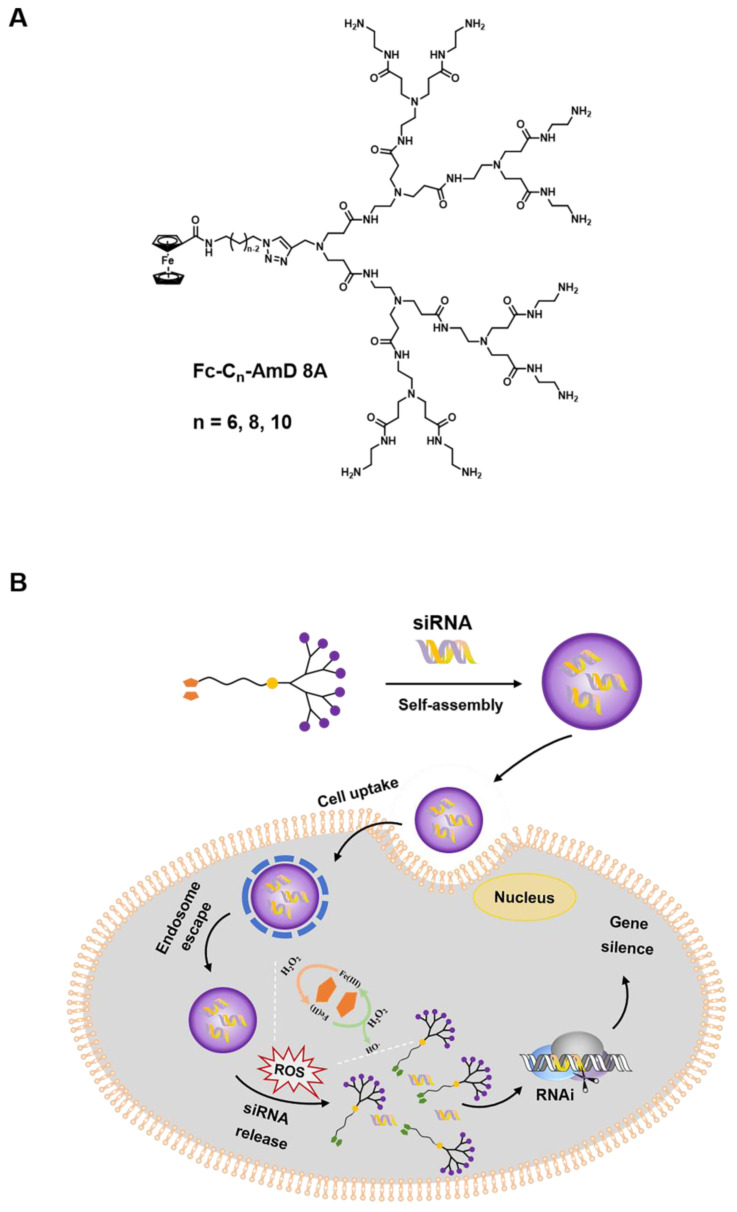
Schematic presentation of ferrocenyl amphiphilic dendrimer-mediated siRNA delivery. (**A**) Chemical structure of the **Fc-AmDs**. (**B**) Cartoon illustration of **Fc-AmD**–mediated siRNA delivery.

Herein, we report a class of ROS–responsive amphiphilic dendrimers (**Fc-AmDs**) for on–demand siRNA delivery upon the exposure to the elevated ROS level in cancer cells (Scheme 1). These dendrimers are composed of hydrophilic PAMAM dendron and hydrophobic alkyl chains with different chain lengths containing ROS–sensitive ferrocene groups (Scheme 1A). The hydrophilic PAMAM dendrons, which feature with amine groups and are positively charged at physiological pH, have been designed to interact with the negatively charged siRNA through electrostatic interactions. Meanwhile, the tertiary amine groups presented in PAMAM dendrons can be further protonated in acidic environments (e.g., pH 5.0 in endosome), thus promoting endosome escape via the proton sponge effect [15,25,26]. Moreover, the hydrophobic alkyl chains with ferrocene groups endow **Fc-AmDs** with good assembly properties. Particularly, the hydrophobic ferrocene is converted to the hydrophilic ferrocenium cation triggered by elevated ROS in cancer cells [27,28]. This hydrophobic–to–hydrophilic transition is anticipated to disrupt the hydrophobic/hydrophilic balance of **Fc-AmD** nanoassemblies and cause their complete disintegration, eventually initiating siRNA release for effective gene silencing within cancer cells (Scheme 1B).

## 2. Materials and Methods

### 2.1. Materials

The human AKT2 siRNA (forward primer: 5′–GCUCCUUCAUUGGGUACAAdTdT–3′; reverse primer: 5′–UAAUGUGCCCGUCCUUGUCdTdT–3′) and GAPDH (forward primer: 5′–AATCCCATCACCATCTTCCA–3′; reverse primer: 5′–TGGACTCCACGACGTACTCA–3′) were purchased from GenScript Biotech Corp. (Nanjing, China). The scramble siRNA (forward primer: 5′–ACGUGACACGUUCGGAGAAdTdT–3′; reverse primer: 5′–UUCUCCGAACGUGUCACGUdTdT–3′) was purchased from Biosyntech Co., Ltd. (Suzhou, China). All the biochemical reagents are suitable for cell culture and were purchased from Sigma–Aldrich (Shanghai, China), Servicebio (Wuhan, China), Cell signaling Technology (Danvers, MA, USA), Vazyme Biotech Co., Ltd. (Nanjing, China) and Thermo Fisher Scientific Inc. (Carlsbad, CA, USA). All the reagents were used without any further purification from commercial sources.

All the chemical reagents are analytical grade and were purchased from Aladdin Ltd. (Shanghai, China), Energy Chemical Ltd. (Shanghai, China), J&K Scientific (Beijing, China) or Sinopharm Chemical Reagent Co., Ltd. (Shanghai, China). Methyl acrylate and ethylenediamine were distilled before use. The compounds **8E** and **8A** were synthesized according to the reported literature [17]. All the other chemicals were purchased from commercial sources and used as received. All the solvents used were freshly distilled. Dialysis tubes were purchased from Yuanye Bio–Technology Co., Ltd. (Shanghai, China).

A Bruker AC (300 MHz) spectrometer was employed to determine the ^1^H NMR and ^13^C NMR spectra of compounds. All the chemical shifts were recorded in parts per million (δ, ppm) with reference to tetramethylsilane (TMS). Mass spectra were measured by using the Agilent 6230 time–of–flight LC/MS (LC/TOF) system. The infrared spectra were recorded by using a Bruker ALPHA FT–IR spectrophotometer (Bruker, Ettlingen, Germany) in the range of 400–4000 cm^−1^.

### 2.2. Synthesis and Characterization of **Fc-AmDs**

The synthetic protocols of **Fc-AmDs** are detailed in Supplementary Materials.

**Fc-C_6_-AmD 8A**: ^1^H NMR (300 MHz, CD_3_OD/CDCl_3_) δ 7.89 (s, 1H), 4.79 (br, 2H), 4.47–4.34 (m, 4H), 4.18 (s, 5H), 3.81 (s, 2H), 3.50–3.15 (m, 30H), 2.96–2.67 (m, 44H), 2.66–2.50 (m, 12H), 2.49–2.25 (m, 28H), 2.05–1.89 (m, 2H), 1.67–1.55 (m, 2H), 1.52–1.34 (m, 4H). ^13^C NMR (75 MHz, CD_3_OD/CDCl_3_) δ 174.04, 173.36, 173.18, 171.93, 143.24, 123.85, 75.58, 70.43, 69.54, 68.05, 52.12, 49.81, 49.25,47.87, 40.39, 39.26, 37.26, 33.47, 30.05, 29.48, 29.01, 28.79, 26.70, 26.23. ESI–HRMS (*m/z*): calcd for C_90_H_167_FeN_33_O_15,_ [M+2H]^2**+**^ 1004.6424, found 1004.6393. HPLC (RT = 16.1 min). IR (cm^−1^): *υ* 1630.50 and 1542.97 (–NH(CO)–).

**Fc-C_8_-AmD 8A**: ^1^H NMR (300 MHz, CD_3_OD/CDCl_3_) δ 7.87 (s, 1H), 4.79 (br, 2H), 4.46–4.33 (m, 4H), 4.18 (s, 5H), 3.82 (s, 2H), 3.47–3.14 (m, 30H), 2.98–2.69 (m, 44H), 2.68–2.53 (m, 12H), 2.49–2.28 (m, 28H), 1.98–1.86 (m, 2H), 1.67–1.52 (m, 2H), 1.45–1.27 (m, 8H). ^13^C NMR (75 MHz, CD_3_OD/CDCl_3_) δ 173.86, 173.36, 173.17, 171.93, 143.35, 123.84, 75.56, 70.42, 69.52, 68.05, 52.12, 49.80, 49.05, 47.68, 41.26, 40.61, 39.10, 37.26, 33.46, 30.00, 29.31, 26.13, 25.97. ESI–HRMS (*m/z*): calcd for C_92_H_171_FeN_33_O_15_, [M+2H]^2**+**^ 1018.6581, found 1018.6541. HPLC (RT = 19.1 min). IR (cm^−1^): *υ* 1631.17 and 1541.60 (–NH(CO)–).

**Fc-C_10_-AmD 8A**: ^1^H NMR (300 MHz, CD_3_OD/CDCl_3_) δ 7.87 (s, 1H), 4.79(br, 2H), 4.44–4.32 (m, 4H), 4.19 (s, 5H), 3.82 (s, 2H), 3.45–3.16 (m, 30H), 2.93–2.68 (m, 44H), 2.66–2.53 (m, 12H), 2.50–2.16 (m, 28H), 1.97–1.85 (m, 2H), 1.66–1.53 (m, 2H), 1.46–1.23(m, 12H). ^13^C NMR (75 MHz, CD_3_OD/CDCl_3_): δ 175.52, 174.94, 145.36, 125.29, 72.07, 71.26, 69.78, 54.08, 51.77, 43.41, 42.54, 42.54, 40.90, 39.13, 35.48, 35.25, 31.68, 31.21, 31.04, 31.04, 30.65, 30.43, 28.40, 27.71. ESI-HRMS (*m/z*): calcd for C_94_H_175_FeN_33_O_15_, [M+2H]^2**+**^ 1032.6737, found 1032.6722. HPLC (RT = 20.2 min). IR (cm^−1^): *υ* 1630.66 and 1542.24 (–NH(CO)–).

### 2.3. HPLC of **Fc-AmDs**

A high–performance liquid chromatography (HPLC) analysis was conducted on a Waters Empower system (Waters 1525, binary HPLC pump, Waters Corp., The Capricorn, Singapore) equipped with a photodiode array detector (Waters 2998, Waters Corp., The Capricorn, Singapore) and a SinoChrom C8 column (5 μm, 4.6 mm × 250 mm). A gradient elution mode of 10–50% acetonitrile in water was employed over 40 min. The mobile phases consisted of acetonitrile and water, both of which contained 0.04% TFA. The flow rate was 0.8 mL/min, with an injection volume of 20 μL. Peaks were detected at 210 nm. The retention times (RTs) were recorded in minutes. All reagents were HPLC–gradient grade and purchased from Anhui Tedia High Purity Solvents Co., Ltd. (Anqing, China) and Aladdin Ltd. (Shanghai, China).

### 2.4. Critical Aggregation Concentration (CAC)

CAC was determined using pyrene as a fluorescent probe. The solutions of **Fc-AmDs** at different concentrations varying from 1.0 × 10^−7^ to 2.0 × 10^−4^ mol/L were prepared, and the final pyrene concentration was 3 × 10^−7^ mol/L in water. The solutions were sonicated for 30 min and kept at room temperature for 2 h to promote the micelle formation prior to fluorescence measurement. Fluorescence spectra were recorded at the emission wavelength of 334 nm on FL8500 fluorescence spectrophotometer at room temperature. The excitation bandwidth was 20 nm, and the emission bandwidth was 1 nm. The fluorescence intensity ratio of I_373_/I_384_ was analyzed as a function of dendrimer concentration.

### 2.5. Potentiometric pH Titration

The solution of **Fc-AmDs** (5 mg in 5 mL) was adjusted to a pH of around 2–3 with 1.0 M HCl. The pH titration was performed with 0.05 M NaOH using METTLER TOLEDO FE28–Bio pH meter (Mettler–Toledo International Inc., Shanghai, China).

### 2.6. ROS Response Measurement of **Fc-AmDs**

The oxidation process of **Fc-AmDs** was detected using UV–Vis spectrophotometry. **Fc-AmDs** were added to H_2_O_2_–containing acetate buffer solution and incubated at 37 °C with gentle shaking. Then, the UV absorption of the solution was measured by UV–1900i UV–Vis spectrophotometer (Shimadzu, Kyoto, Japan).

The hydroxyl radical (·OH) produced by Fenton–like reaction was detected using TMB. Briefly, **Fc-AmDs** and TMB were added to H_2_O_2_–containing acetate buffer solution and incubated at 37 °C with gentle shaking. Then, the UV absorption of the mixed solution was measured by UV–Vis spectrophotometry. 

### 2.7. Dynamic Light Scattering (DLS)

#### 2.7.1. DLS Analysis of **Fc-C_8_-AmD 8A**

A 200 μM solution of **Fc-C_8_-AmD 8A** was prepared by dissolving the compound in ultrapure water. The sizes of **Fc-C_8_-AmD 8A** solutions were measured using a NanoBrookOmni (Brookhaven, Holtsville, NY, USA) equipped with a standard 633 nm laser at 25 °C.

#### 2.7.2. DLS Analysis of siRNA/**Fc-C_8_-AmD 8A** Nanoparticles

The solution of siRNA/**Fc-C_8_-AmD 8A** complexes was prepared via mixing the siRNA solution and **Fc-C_8_-AmD 8A** solution at N/P ratio of 10, resulting in a final concentration of 1.0 μM for the siRNA. Ultrapure water was used for the solvent. The sizes of the siRNA/**Fc-C_8_-AmD 8A** complexes solutions were measured by the same DLS method as described above.

#### 2.7.3. DLS Analysis of the ROS–Triggered Disassembly of siRNA/**Fc-C_8_-AmD 8A** Nanoparticles

The solutions of siRNA/**Fc-C_8_-AmD 8A** complexes were prepared as described above. PB solution (pH 5.0) was used for the solvent. The sizes of the siRNA/**Fc-C_8_-AmD 8A** complexes in the absence and presence of H_2_O_2_ were measured at different time points (0, 1, 2, 4, 6, 8, 12, and 24 h) using the same DLS method as described above. 

### 2.8. Gel Retardation Analysis

The solutions of **Fc-AmDs** and siRNA were mixed at different N/P ratios ranging from 0.2 to 10 and incubated at 37 °C for 30 min. The final concentration of siRNA in each sample was 200 ng/well. The siRNA/**Fc-AmDs** complexes were analyzed via electrophoretic mobility–shift assays conducted using 2% agarose gels in standard TAE buffer for 15 min. Following staining with GoodView nucleic acid dyes, the siRNA bands were imaged using an automatic chemiluminescence imaging system (5200 Multi) (Tanon, Shanghai, China). 

### 2.9. Stability of siRNA/**Fc-C_8_-AmD 8A** Complex against RNase

An aliquot containing 200 ng of siRNA and the indicated amounts of **Fc-C_8_-AmD 8A** solution at N/P ratio of 10 was incubated in the presence of RNase A (1.0 μg/mL) at 37 °C for different times (0, 10, 30, 60, 90, and 120 min). Afterwards, aliquots (10.0 μL) of the corresponding solution were added 1.0 μL of 2% SDS solution on ice. The mixtures were analyzed by gel retardation as described above. Naked siRNA served as a control. 

### 2.10. RNA Dissociation Assay

A solution containing 0.1 μg ethidium bromide (EB), 1.32 μg siRNA, and an appropriate amount of **Fc-AmDs** at a N/P ratio of 10 in PB buffer at pH 5.0 was added to the black 96–well plate and incubated at 25 °C for 30 min. Then, to 60 μL of the above siRNA/**Fc-AmDs** complexes was added the 40 μL of heparin solution in PB at different concentrations varying from 0 to 50 U/mL to achieve a total volume of 100 μL. After a further incubation of 30 min at 25 °C, the mixture was measured at 590 nm using a Cytation5 Microplate Reader (BioTek, Winooski, VT, USA) with 360 nm fluorescence excitation. The fluorescence values were normalized to wells containing EB siRNA solution only. All samples were assayed in triplicate. 

### 2.11. Cell Culture

Mouse fibroblast L929 cells, madin-darby canine kidney MDCK cells, human normal liver L02 cells, and human ovarian cancer SKOV–3 cells were purchased from Tongpai Biotechnology Co., Ltd. (Shanghai, China). L929 cells were maintained in DMEM (Hyclone, Logan, UT, USA), supplemented with 10% FBS. MDCK cells were maintained in MEM (Hyclone, Logan, UT, USA) with 10% FBS. Human ovarian cancer SKOV–3 cells were cultured in McCOY’S 5A (Hyclone, Logan, UT, USA) with 10% FBS. L02 cells were maintained in RMPI–1640 (Hyclone, Logan, UT, USA) with 10% FBS. All cells were cultured in an incubator with a humidified environment of 5% CO_2_ and a constant temperature of 37 °C. 

### 2.12. MTT(3–(4,5–Dimethylthiazol–2–yl)–2,5–Diphenyltetrazolium Bromide) Assay

Cancer cells (SKOV–3 cells) and normal cells (MDCK cells, L929 cells, and L02 cells) (8 × 10^3^) were seeded in 96–well plates and cultured for 24 h. Cells were then treated with **Fc-AmDs** at different concentrations, ranging from 0.25 to 50 μM, for 8 h. After transfection, the medium was replaced with fresh medium. Then, 48 h later, the cells were treated with MTT solution and incubated for a further 4 h. After removing the solution, the cells were re–suspended in DMSO. Cytation5 (BioTek, Winooski, VT, USA) was used to measure the optical density (OD) of DMSO solutions, which was read at 570 nm. The viability of cells was assessed by the difference between the OD values of treated and untreated cells. All samples were assayed in triplicate. 

### 2.13. Flow Cytometry

One day prior to the transfection, SKOV–3 cells were seeded at 8.0 × 10^4^ cells/well in a 24–well plate. The cells were incubated with Cy5–labeled siRNA/**Fc-C_8_-AmD 8A** complex (50 nM Cy5–siRNA, N/P ratio 10) for 1, 2, 4, 6, and 8 h. After three washes with cold PBS, the cells were analyzed by flow cytometry (Attune NxT, Thermo Fisher Scientific). All samples were assayed in triplicate. 

### 2.14. Confocal Microscopy

One day prior to the transfection, SKOV–3 cells were seeded in 2.5 dishes (8.0 × 10^4^ cells/dish). The cells were incubated with Cy5–labeled siRNA/**Fc-C_8_-AmD 8A** complex (50 nM Cy5–siRNA, N/P ratio of 10) for 1, 2, 4, 6, and 8 h at 37 °C. After three washes with cold PBS, the cells were stained by PBS mixed with Hoechst33342 (10 μg/mL) and Lyso-Tracker Red (0.10 μM) for 10 min at 37 °C. Images were acquired with a Zeiss LSM880 Meta laser scanning confocal microscope (Carl Zeiss, Jena, Germany) using ZEN2.3 pro software (Carl Zeiss GmbH). 

### 2.15. ROS Level Measurement

SKOV–3 and NAC–treated SKOV–3 cells (1.5 × 10^5^ cells/group) were treated with the CellROX^®^ orange Reagent (Thermo Fisher Scientific, Carlsbad, CA, USA) at a final concentration of 1.0 μM and incubated at 37 °C for 30 min. Following three washes with PBS, the mean fluorescence intensity of the cells was quantified by flow cytometry (Attune NxT, Thermo Fisher Scientific). Each assay was performed in triplicate. 

### 2.16. In Vitro Transfection

SKOV–3 cells and NAC–pretreated SKOV–3 cells were seeded in 6–well plates and grown for 24 h. The solutions of siAKT2/**Fc-AmDs** complexes with 50 nM siRNA at an N/P ratio of 10 were prepared before transfection. After incubation with siAKT2/**Fc-AmDs** complexes for 8 h, the transfection mixture was replaced with the complete medium. And then, the cells were incubated for an additional 48 h for qRT–PCR assay and 72 h for Western blot assay. 

#### 2.16.1. Effect of Dioleoylphosphatidylethanolamine (DOPE)

The effect of DOPE was assessed by the transfection experiments as described above. Before transfection, the solutions of siAKT2/**Fc-C_8_-AmD 8A** complexes were prepared with DOPE at mole ratio of 1.0 (DOPE/dendrimer). 

#### 2.16.2. Effect of Bafilomycin A1

After preincubation of SKOV–3 cells with 200 nM bafilomycin A1 at 37 °C for 1 h, the effect of bafilomycin A1 was assessed by the transfection experiments as described above. 

### 2.17. Quantitative Real-Time (qRT)–PCR Analysis

The transfection experiments were performed in SKOV–3 cells as described above. After isolation with the Trizol method (Vazyme Biotech Co., Ltd., Nanjing, China), the total RNAs of SKOV–3 cells were reverse–transcribed by a Reverse Transcription Kit (Vazyme Biotech Co., Ltd., Nanjing, China). The expression of AKT2 was analyzed by quantitative real–time PCR performed on the QuantStudio3™ real–time PCR System (Applied Bisystems, Thermo Fisher Scientific) using 2 × SYBR Green (Vazyme Biotech Co., Ltd., Nanjing, China). The expression of GAPDH was used for normalization of the qPCR data. 

### 2.18. Western Blot Analysis

Samples containing equal amounts of protein (15 μg) from SKOV–3 cells were separated by SDS–PAGE gradient gel and transferred to the PVDF membrane after electrophoresis. After being blocked in 5% skimmed milk, the PVDF membranes were incubated with anti–human vinculin rabbit polyclonal antibody (Cell signaling Technology, Danvers, MA, USA, 1:5000) or anti–human AKT2 rabbit polyclonal antibody (Cell signaling Technology, Danvers, MA, USA, 1:1000) at 4 °C for 12 h. Then, the membranes were washed and incubated with anti–rabbit or anti-mouse monoclonal secondary antibodies (Invitrogen, Boston, MA, USA, 1:5000) for 2 h at 25 °C. Specific proteins were recorded by an enhanced-chemiluminescence Western blotting analysis system (Tanon, Shanghai, China). 

### 2.19. Statistical Tests

All data are presented as mean ± SD unless otherwise indicated. One–way analysis of variance (ANOVA), two–way ANOVA, unpaired Student’s *t*-test or Mann–Whitney test (GraphPad Prism 8.01) were used for statistical analysis. A *p* value < 0.05 was considered statistically significant, whereby all significant values in various figures are indicated as follows: * *p* ≤ 0.05; ** *p* ≤ 0.01; *** *p* ≤ 0.001. 

## 3. Results and Discussions

### 3.1. Ferrocenyl Amphiphilic Dendrimer Synthesis

**Scheme 2:**
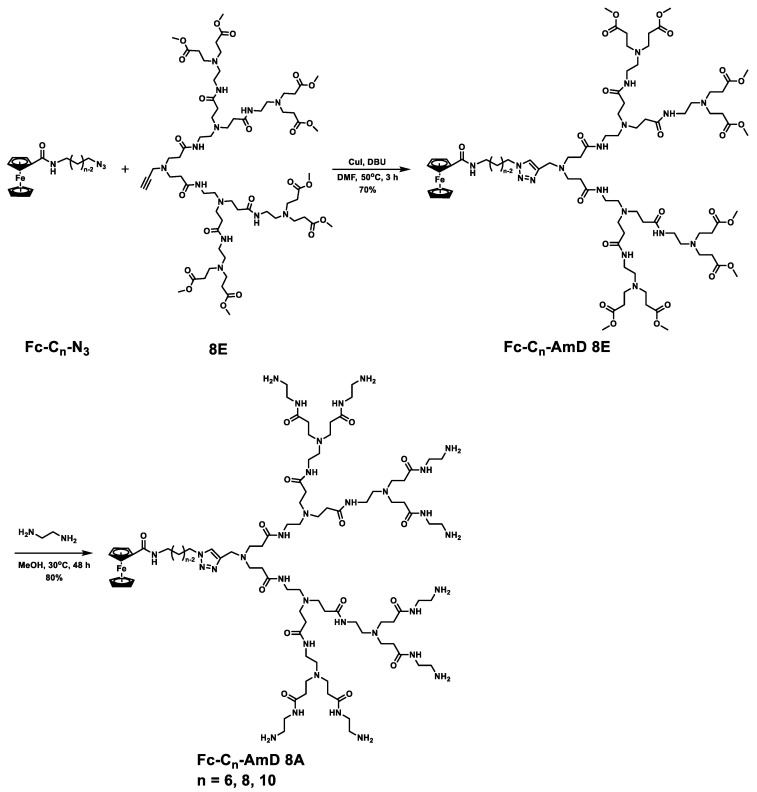
Synthesis routes of **Fc-AmDs**.

Ferrocenyl amphiphilic dendrimers, denoted as **Fc-AmDs**, were prepared according to the synthetic routes shown in Scheme 2 and Scheme S1. Briefly, haloalkanols or dihaloalkanes were used as the starting materials and followed by Gabriel amine synthesis, azidation, and amidation with ferrocenecarboxylic acids to build the ferrocene and azido–bearing hydrophobic alkyl chains **Fc-C_n_-N_3_** with different chain lengths (Scheme S1). Then, the obtained **Fc-C_n_-N_3_** were coupled with the alkynyl–bearing PAMAM dendron **8E** by the copper(I)–catalyzed azide–alkyne cycloaddition (CuAAC) click reaction to generate the ester–terminated dendrimer conjugates **Fc-C_n_-AmD 8E**, respectively. These conjugates were further subjected to amidation with ethylenediamine to yield the desired ferrocenyl amphiphilic dendrimers **Fc-C_n_-AmD 8A** (n = 6, 8, 10, Scheme 2). The structural integrities and purities of all the obtained dendrimers were characterized and confirmed by ^1^H–NMR, ^13^C–NMR, electrospray high–resolution mass spectrometry (ESI–HRMS), infrared spectrum (IR), and high–performance liquid chromatography (HPLC) (Figures S1–S3 and Table S1). Moreover, all established dendrimers **Fc-C_n_-AmD 8A** harbor tertiary amines in the interior and primary amines at the surface, with p*K_a_* of about 5 and 9, respectively, as revealed by pH titration (Figure S4). Therefore, the primary amines in these dendrimers are able to protonate at physiological pH 7.4, while the tertiary amines can be further protonated under acidic conditions (pH 5.0), implying that they have a buffering effect [29]. 

### 3.2. ROS Response of **Fc-AmDs**

**Figure 1:**
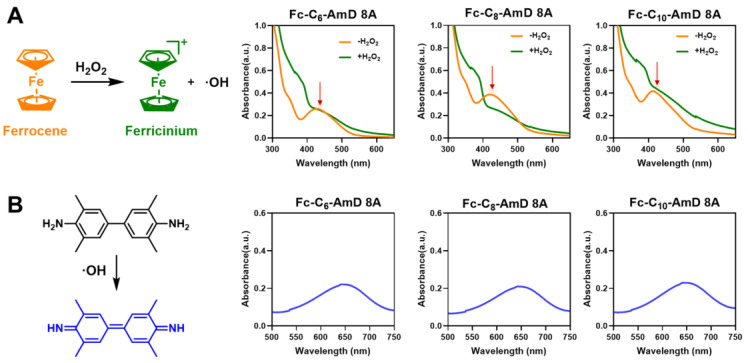
Characterization of the ROS–responsive properties of **Fc-AmDs**. (**A**) UV–vis absorption spectra of **Fc-AmDs** before and after hydrogen peroxide (H_2_O_2_) oxidation; (**B**) the hydroxyl radicals produced by the Fenton reaction of **Fc-AmDs** with H_2_O_2_ using TMB by UV–vis spectrophotometer.

After obtaining ferrocenyl amphiphilic dendrimers (**Fc-AmDs**), we wished to study their ROS–responsive characteristics under simulated ROS conditions (in the presence of H_2_O_2_). As a typical Fenton reagent, the ferrocenyl group is prone to a Fenton–like reaction upon treatment with H_2_O_2_, which generates oxidized ferrocenium and hydroxyl radicals (**·**OH) [27]. Thus, we incubated **Fc-AmDs** with H_2_O_2_ and found that the characteristic UV absorptions (430–450 nm) originating from the ferrocenyl groups rapidly disappeared (Figure 1A). This result indicated that the ferrocene moieties in **Fc-AmDs** indeed undergo a Fenton–like reaction and change into their oxidative forms following exposure to H_2_O_2_. Moreover, hydroxyl radicals (**·**OH) produced by the Fenton reaction can be detected by 3,3′,5,5′–tetramethylbenzidine (TMB), as it can be oxidized by hydroxyl radicals (**·**OH) to oxTMB, which has a characteristic UV absorption at around 650 nm [30]. Therefore, we added TMB after exposing **Fc-AmDs** to H_2_O_2_. The appearance of the characteristic UV absorptions from oxTMB at around 650 nm further confirmed the production of ·OH during the oxidation of ferrocenyl groups in **Fc-AmDs** (Figure 1B and Figure S5). These findings suggest that the ferrocene–bearing **Fc-AmDs** indeed can undergo Fenton–like reactions under ROS conditions and, hence, possess the potential for on–demand drug delivery in response to ROS.

### 3.3. Best Performance of **Fc-AmDs** in siRNA Delivery and the Underlying Rationale

**Figure 2:**
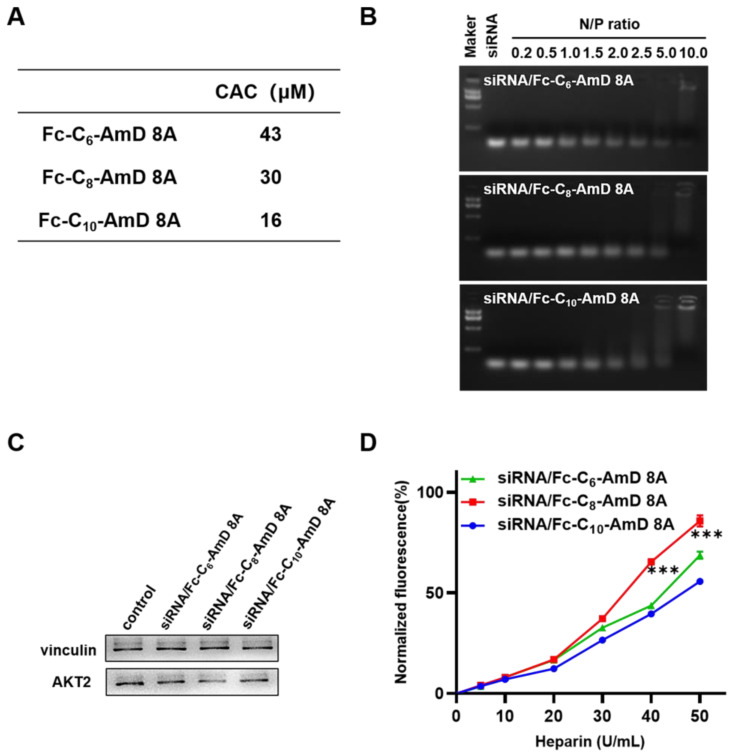
(**A**) The CAC values of the **Fc-AmDs** were determined using fluorescent probe pyrene. (**B**) Gel retardation assay of siRNA with **Fc-AmDs** at N/P ratios ranging from 0.2 to 10 (200 ng siRNA per well). (**C**) AKT2 protein expression on SKOV–3 cells determined by Western blotting after treating with siAKT2/**Fc-AmDs** complexes (50 nM siRNA, N/P ratio of 10). (**D**) siRNA release from the siRNA/**Fc-AmDs** complexes was determined by heparin replacement assay (1.32 μg siRNA per well, N/P ratio of 10). *** *p* ≤ 0.001 (mean ± SD, n = 3).

The obtained ferrocenyl amphiphilic dendrimers, **Fc-AmDs**, are speculated to be able to self–assemble in aqueous solution by virtue of their amphiphilic nature. Therefore, we investigated the self–assembly behavior of **Fc-AmDs** by measuring the value of their critical aggregation concentration (CAC) using hydrophobic fluorescent probe pyrene. The detected CAC values of **Fc-C_6_-AmD 8A**, **Fc-C_8_-AmD 8A**, and **Fc-C_10_-AmD 8A** were 43, 30, and 16 μmol/L, respectively (Figure 2A and Figure S6), confirming the inverse relationship between the CAC values of **Fc-AmDs 8A** and the length of their hydrophobic portions, i.e., **Fc-C_10_-AmD 8A** bearing the longest hydrophobic chain self-assembles and packs most efficiently among these three dendrimers.

We next examined their ability to bind siRNA at varying N/P ratios using agarose gel electrophoresis migration assays. As shown in Figure 2B, all three **Fc-AmDs** can efficiently bind siRNA and completely retard the migration of siRNA at an N/P ratio of 10, while **Fc-C_10_-AmD 8A** exhibited relatively better binding ability than other two dendrimers and started to retard the migration of siRNA from an N/P ratio of 2.0. These results denoted that **Fc-C_10_-AmD 8A**, with the best self–assembly capability, achieved siRNA binding most efficiently.

Prior to assessing the siRNA delivery capacity of **Fc-AmDs**, we first examined their safety, which is an important parameter for developing siRNA delivery vectors. The 3– (4,5–dimethylthiazol–2–yl)–2,5–diphenyltetrazolium bromide (MTT) assay was used to detect metabolic toxicity by measuring cell viability. As shown in Figure S7 and Table S2, all **Fc-AmDs** exhibited no significant influence on the proliferation of all the tested cell lines, including mouse fibroblast L929 cells, human–derived normal liver L02 cells, Madin–Darby canine kidney cells, and ovarian cancer SKOV–3 cells. These results indeed confirmed that all the three **Fc-AmDs** are devoid of any notable toxicity.

Encouraged by the favorable safety profile of **Fc-AmDs**, we then evaluated their ability to deliver siRNA targeting AKT2 in SKOV–3 cells. AKT2 is a proto–oncogene involved in the PI3K/AKT/mTOR signaling pathway, and its amplification contributes to tumorigenesis, cancer cell proliferation, invasion, metastasis and angiogenesis, even to protecting cancer cells from drug–induced apoptosis [31,32]. Therefore, AKT2 is recognized as an important therapeutic target in ovarian cancer. Among the ferrocenyl amphiphilic dendrimers, dendrimer **Fc-C_8_-AmD 8A** exhibited effective gene silencing with AKT2 siRNA (siAKT2), whereas no significant gene silencing was observed after siAKT2 delivered by the other two ferrocenyl amphiphilic dendrimers carrying the shortest or longest alkyl chains (**Fc-C_6_-AmD 8A** or **Fc-C_10_-AmD 8A**) (Figure 2C and Figure S8).

In order to understand why **Fc-C_8_-AmD 8A** performed better in siRNA delivery than the other two **Fc-AmDs**, we further studied the release of siRNA from the corresponding siRNA/dendrimer complexes using the heparin replacement assay. Heparin is a kind of highly negatively charged polysaccharide that can compete with siRNA for binding to dendrimers. As the concentration of heparin increased, the siRNA molecules dissociated more efficiently from their complex with **Fc-C_8_-AmD 8A** than from their complexes formed with the two other dendrimers (**Fc-C_6_-AmD 8A** and **Fc-C_10_-AmD 8A**) (Figure 2D). This finding confirmed that the siRNA release process is indeed more efficient for siRNA/**Fc-C_8_-AmD 8A** than for siRNA/**Fc-C_6_-AmD 8A** and siRNA/**Fc-C_10_-AmD 8A**.

Further investigations showed that the average sizes of empty **Fc-C_8_-AmD 8A** and siRNA/**Fc-C_8_-AmD 8A** complexes measured by dynamic light scattering were approximately 100 and 70 nm, respectively. The smaller size distribution of the siRNA/**Fc-C_8_-AmD 8A** complexes (Figure S9) indicated that the **Fc-C_8_-AmD 8A** was able to compact siRNA into stable nanoparticles. Such a nanoscale complex indeed can effectively protect siRNA from enzymatic degradation (Figure S10), facilitate efficient uptake of the siRNA cargos by cells (Figure S11), and release the loaded siRNA at its final destination.

Collectively, these findings demonstrate that the proper balance of the hydrophobic chain length and the hydrophilic dendritic component of **Fc-AmDs** is crucial for their self-assembly behavior, which in turn plays an important role in their performance in siRNA delivery. One of these amphiphilic dendrimers, **Fc-C_8_-AmD 8A**, possesses an optimal balance of hydrophobicity and hydrophilicity and is, therefore, able to generate stable nanosized assemblies for optimal siRNA binding and the most efficient siRNA release. These properties make **Fc-C_8_-AmD 8A** outperform in siRNA delivery and gene silencing compared to the two other dendrimers (**Fc-C_6_-AmD 8A** and **Fc-C_10_-AmD 8A**).

### 3.4. Specific ROS–Responsive siRNA Delivery Mediated by **Fc-C_8_-AmD 8A**

**Figure 3:**
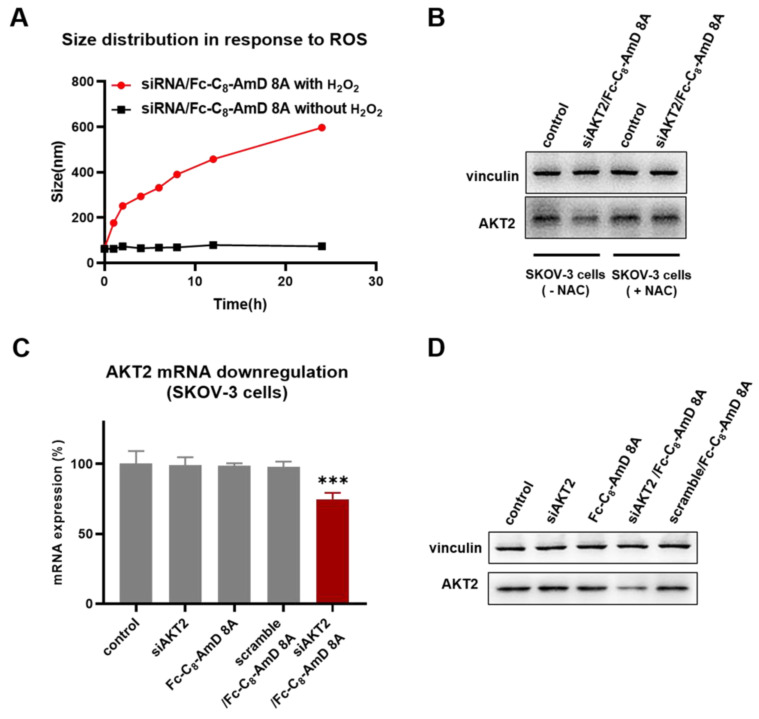
(**A**) DLS analysis of the ROS–responsive disassembly of siRNA/**Fc-C_8_-AmD 8A** nanoparticles (1.0 μM siRNA, N/P ratio of 10). (**B**) ROS–responsive siRNA delivery mediated by **Fc-C_8_-AmD 8A** in ROS–rich SKOV–3 cells and ROS–poor SKOV–3 cells (pretreated with antioxidant NAC) (50 nM siRNA, N/P ratio of 10). (**C**) mRNA and (**D**) protein expression of AKT2 determined by qRT–PCR and Western blot after treating with **Fc-C_8_-AmD 8A** with siAKT2 in comparison with control, siAKT2 alone, **Fc-C_8_-AmD 8A** alone, **Fc-C_8_-AmD 8A** with scrambled siRNA (50 nM siRNA, N/P ratio of 10). *** *p* ≤ 0.001 (mean ± SD, n = 3).

Benefiting from the optimal siRNA delivery performance of **Fc-C_8_-AmD 8A** alongside its ROS–responsive characteristics, it is expected to be able to achieve on–demand delivery of siRNA therapeutics to cancer cells that typically have high levels of ROS. First, the specific disassembly of siRNA/**Fc-C_8_-AmD 8A** nanoparticles under high ROS conditions in cancer cells is a prerequisite for on–demand delivery of siRNA. We, thus, studied the siRNA/**Fc-C_8_-AmD 8A** nanoparticles in the presence of H_2_O_2_, simulating the high ROS conditions in cancer cells. As revealed by DLS analysis, the size of the siRNA/**Fc-C_8_-AmD 8A** complex was increased from the original value of supposedly 70 nm to over 600 nm following the exposure to H_2_O_2_. This result confirms that the siRNA/**Fc-C_8_-AmD 8A** complex becomes loose due to the good ROS–responsive characteristics of **Fc-C_8_-AmD 8A**, which will facilitate the release of loaded siRNA within the complex. (Figure 3A). Furthermore, **Fc-C_8_-AmD 8A**-mediated delivery of siAKT2 was indeed specific to ROS–rich ovarian cancer SKOV-3 cells, as a significantly decreased AKT2 gene silencing was observed in N–Acetyl–L–cysteine (NAC) pretreated SKOV–3 cells (Figure 3B and Figure S12). NAC, an antioxidant, has been demonstrated to significantly reduce the ROS levels in SKOV–3 cells (Figure S13).

Moreover, following **Fc-C_8_-AmD 8A**–mediated delivery of siAKT2, the expression of AKT2 was significantly and specifically suppressed at both mRNA and protein levels in ROS–rich SKOV3 cells, whereas no detectable gene silencing was observed with the treatment of either **Fc-C_8_-AmD 8A** or siRNA alone or with a scrambled siRNA/**Fc-C_8_-AmD 8A** complex. Altogether, these results suggest that **Fc-C_8_-AmD 8A** enables efficient delivery of siRNA therapeutics to cancer cells and achieves potent gene silencing in response to high ROS content, which is in line with our design concept for **Fc-C_8_-AmD 8A** as a ROS–responsive delivery vehicle.

### 3.5. **Fc-C_8_-AmD 8A** Capitalizes on the Delivery Advantage of Both Lipid and Dendrimer Vectors

**Figure 4:**
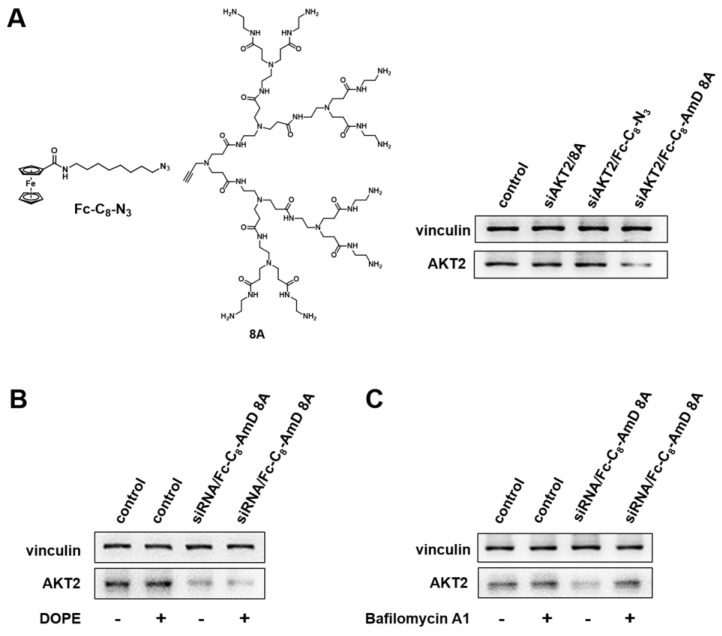
**Fc-C_8_-AmD 8A**–mediated siRNA delivery combines the beneficial properties of both lipid and dendrimer vectors. (**A**) Compared to **Fc-C_8_-AmD 8A**, neither the Fc alkyl chain entity **Fc-C_8_-N_3_** nor the dendron **8A** led to any gene silencing with 50 nM siRNA at N/P ratio of 10. (**B**) Dioleoylphosphatidylethanolamine (DOPE) enhanced the gene silencing of AKT2 after treatment of **siRNA/Fc-C_8_-AmD 8A** complexes with 50 nM siRNA at N/P ratio of 10 on SKOV–3 cells. (**C**) The presence of bafilomycin A1 decreased the **Fc-C_8_-AmD 8A**–mediated gene silencing of AKT2 on SKOV–3 on cells (50 nM siRNA, N/P ratio of 10).

Ferrocenyl amphiphilic dendrimer **Fc-C_8_-AmD 8A** is a lipid/dendrimer hybrid. Therefore, it is conceived to take the delivery advantages of both lipid and dendrimer vectors. To confirm this, we evaluated the siRNA delivery efficacy of the alkyl chain containing the ferrocenyl moiety (**Fc-C_8_-N_3_**) and the PAMAM dendron (**8A**) in comparison with **Fc-C_8_-AmD 8A**. As shown in Figure 4A and Figure S14, except for **Fc-C_8_-AmD 8A**, the alkyl chain bearing the Fc moiety **Fc-C_8_-N_3_** and the PAMAM dendron **8A** was unable to produce notable gene silencing, indicating the amphiphilic structure of **Fc-C_8_-AmD 8A** is of great importance for its effective siRNA delivery.

We further assessed the efficacy of siRNA delivered by **Fc-C_8_-AmD 8A** in the presence of dioleoylphosphatidyl–ethanolamine (DOPE). DOPE is a fusogenic lipid that promotes membrane fusion and is often used as a helper lipid to improve lipid–mediated delivery. The addition of DOPE significantly improved the AKT2 silencing achieved by **Fc-C_8_-AmD 8A**–mediated siRNA delivery (Figure 4B and Figure S15), confirming that **Fc-C_8_-AmD 8A** possesses the delivery characteristics of lipid vectors.

We next examined whether **Fc-C_8_-AmD 8A** benefits from the “proton sponge effect” inherited from PAMAM dendrimer vectors, resulting in efficient siRNA delivery. PAMAM dendrimers frequently utilize the “proton sponge effect” to promote endosome escape and subsequent cargo release in the cytoplasm. We therefore verified **Fc-C_8_-AmD 8A**–mediated siRNA delivery in the presence of bafilomycin A1, a proton pump inhibitor that impedes the acidification of endosomes. As presented in Figure 4C and Figure S16, the siRNA delivery and AKT2 silencing efficacy mediated by **Fc-C_8_-AmD 8A** was significantly reduced in the presence of bafilomycin A1, implying **Fc-C_8_-AmD 8A**–mediated siRNA delivery is also critically dependent on the “proton sponge effect”. In addition, the pH titration profile of **Fc-C_8_-AmD 8A** showed that it is able to absorb protons in endosomes (at pH 5.0) via protonation of its interior tertiary amines (Figure S4), further supporting the involvement of the proton sponge effect.

Altogether, these findings demonstrated that **Fc-C_8_-AmD 8A** indeed harnesses the beneficial properties of lipid and dendrimer vectors for effective delivery of siRNA therapeutics.

## 4. Conclusions

In summary, we have successfully established ROS–responsive ferrocenyl amphiphilic dendrimers (**Fc-AmDs**) as on–demand delivery platforms for cancer cell specific siRNA delivery. These **Fc-AmDs** are composed of hydrophobic alkyl chains with ferrocene moieties and hydrophilic PAMAM dendrons, possessing good self–assembly behavior in aqueous solutions, ROS-–responsive properties, and favorable safety profiles. One of these dendrimers, **Fc-C_8_-AmD 8A**, was endowed with the optimal balance between the hydrophobic chain length and the hydrophilic dendron, exhibiting the optimal siRNA binding strength and best siRNA release ability. It was capable of packing siRNA into nanoparticles to protect siRNA and promoting cellular uptake, resulting in the best performance in siRNA delivery. This ferrocenyl amphiphilic dendrimer is also able to harness the features and the delivery advantages of both lipid and dendrimer vectors. Notably, the ROS properties of **Fc-C_8_-AmD 8A** allows efficient disassembly of its complex with siRNA in ROS–rich environments, thereby inducing specific and significant silencing of the AKT2 gene in ovarian cancer SKOV–3 cells. All in all, our results demonstrate that this ferrocenyl amphiphilic dendrimer **Fc-C_8_-AmD 8A** does provide a novel on–demand delivery approach for cancer cell–specific siRNA delivery by exploiting the high levels of ROS in cancer cells. This work opens a new perspective on the design of on-demand drug delivery nanosystems based on self–assembling dendrimers.

## Data Availability

All study data are included within the article and/or Supplementary Materials.

## Supplementary Materials

The following supporting information can be downloaded at https://www.mdpi.com/article/10.3390/pharmaceutics16070936/s1, Scheme S1: Synthetic route of the hydrophobic part; Figures S1–S3: ^1^H and ^13^C NMR, HPLC, ESI–HRMS, and IR spectra of **Fc-C_6_-AmD 8A**, **Fc-C_8_-AmD 8A,** and **Fc-C_10_-AmD 8A**; Figure S4: Potentiometric pH titration profile of **Fc-AmDs**; Figure S5: The ROS–responsive performance of **Fc-C_8_-AmD 8A**; Figure S6: The CAC measurements of **Fc-AmDs**; Figure S7: The MTT assay of the metabolic toxicity of **Fc-AmDs** in MDCK cells, L02 cells, L929 cells, and SKOV–3 cells; Figure S8: AKT2 protein expression on SKOV–3 cells after treatment with siRNA/**Fc-AmDs** complexes quantified by Western blotting; Figure S9: Size distribution of **Fc-C_8_-AmD 8A** and siRNA/**Fc-C_8_-AmD 8A** complexes by intensity determined using dynamic light scattering analysis; Figure S10: Agarose gel retardation of siRNA/**Fc-C_8_-AmD 8A** complexes at different times after incubation with RNase A and SDS (200 ng siRNA/well, N/P ratio of 10); Figure S11: (A) Flow cytometry analysis and (B) confocal imaging of the cell uptake of the siRNA/**Fc-C_8_-AmD 8A** complexes in SKOV–3 cells (50 nM Cy5–labeled siRNA, N/P ratio of 10). Red: Cy5–labeled siRNA; Blue: Hoechst33342 labeling nuclei, Scale 20 μm; Figure S12: ROS–responsive siRNA delivery mediated by **Fc-C_8_-AmD 8A** in ROS-rich SKOV–3 cells and ROS–poor SKOV–3 cells (pretreated with antioxidant NAC) (50 nM siRNA, N/P ratio of 10). *** *p* ≤ 0.001 (mean ± SD, n = 3); Figure S13: ROS levels in human ovarian cancer SKOV–3 cells and SKOV–3 cells pretreated with the antioxidant N–acetyl–cysteine (NAC) (10 mM) quantified using CellROX orange reagent by flow cytometry. *** *p* ≤ 0.001 (mean ± SD, n = 3); Figure S14: Gene silencing following siAKT2 delivery mediated by **8A, Fc-C_8_-N_3_,** and **Fc-C_8_-AmD 8A** (50 nM siRNA, N/P ratio of 10). *** *p* ≤ 0.001 (mean ± SD, n = 3); Figure S15: Dioleoylphosphatidylethanolamine (DOPE) enhanced the gene silencing of AKT2 after treatment of siRNA/**Fc-C_8_-AmD 8A** complexes with 50 nM siRNA at N/P ratio 10 on SKOV–3 cells. *** *p* ≤ 0.001 (mean ± SD, n = 3); Figure S16: AKT2 protein downregulation following treatment with the siAKT2/**Fc-C_8_-AmD 8A** complex (50 nM siRNA, N/P ratio of 10) on SKOV–3 cells in the presence and absence of the proton pump inhibitor, bafilomycin A1. *** *p* ≤ 0.001 (mean ± SD, n = 3); Table S1: The information on **Fc-AmDs** from HPLC detection; Table S2: The IC_50_ of **Fc-AmDs** was assessed by MTT assay on normal and cancer cells.
