# Supplementary material for: ROS–Responsive Ferrocenyl Amphiphilic PAMAM Dendrimers for On–Demand Delivery of siRNA Therapeutics to Cancer Cells
Source: Pharmaceutics. 2024 Jul 13;16(7):936. doi: 10.3390/pharmaceutics16070936 (PMC11280363; doi:10.3390/pharmaceutics16070936)
Supplement: Supplementary file 1 [file pharmaceutics-16-00936-s001.zip › pharmaceutics-3025647-supplementary.pdf]

pharmaceutics

MDPI

Supplementary Materials: ROS–Responsive Ferrocenyl Amphiphilic PAMAM Dendrimers for On–Demand Delivery of siRNA Therapeutics to Cancer Cells

Peng Chen, Zhihui Wang, Xinmo Wang, Junni Gong, Ju Sheng, Yufei Pan, Dandan Zhu and Xiaoxuan Liu

Table of Content

Supporting Information for .... Error! Bookmark not defined.

Scheme S1 1

Figure S1 .... Error! Bookmark not defined.

Figure S2 .... Error! Bookmark not defined.

Figure S3 .... Error! Bookmark not defined.

Figure S4 .... Error! Bookmark not defined.

Figure S5 .... Error! Bookmark not defined.

Figure S6 .... Error! Bookmark not defined.

Figure S7 .... Error! Bookmark not defined.

Figure S8 .... Error! Bookmark not defined.

Figure S9 .... Error! Bookmark not defined.

Figure S10 12

Figure S11 .... Error! Bookmark not defined.

Figure S12 .... Error! Bookmark not defined.

Figure S13 .... Error! Bookmark not defined.

Figure S14 .... Error! Bookmark not defined.

Figure S15 .... Error! Bookmark not defined.

Figure s16 .... Error! Bookmark not defined.

Table S1 .... Error! Bookmark not defined.

Table S2 .... Error! Bookmark not defined.

Synthesis and characterization of ferrocenyl amphiphilic dendrimers 15

Scheme S1. Synthetic route of the hydrophobic part.

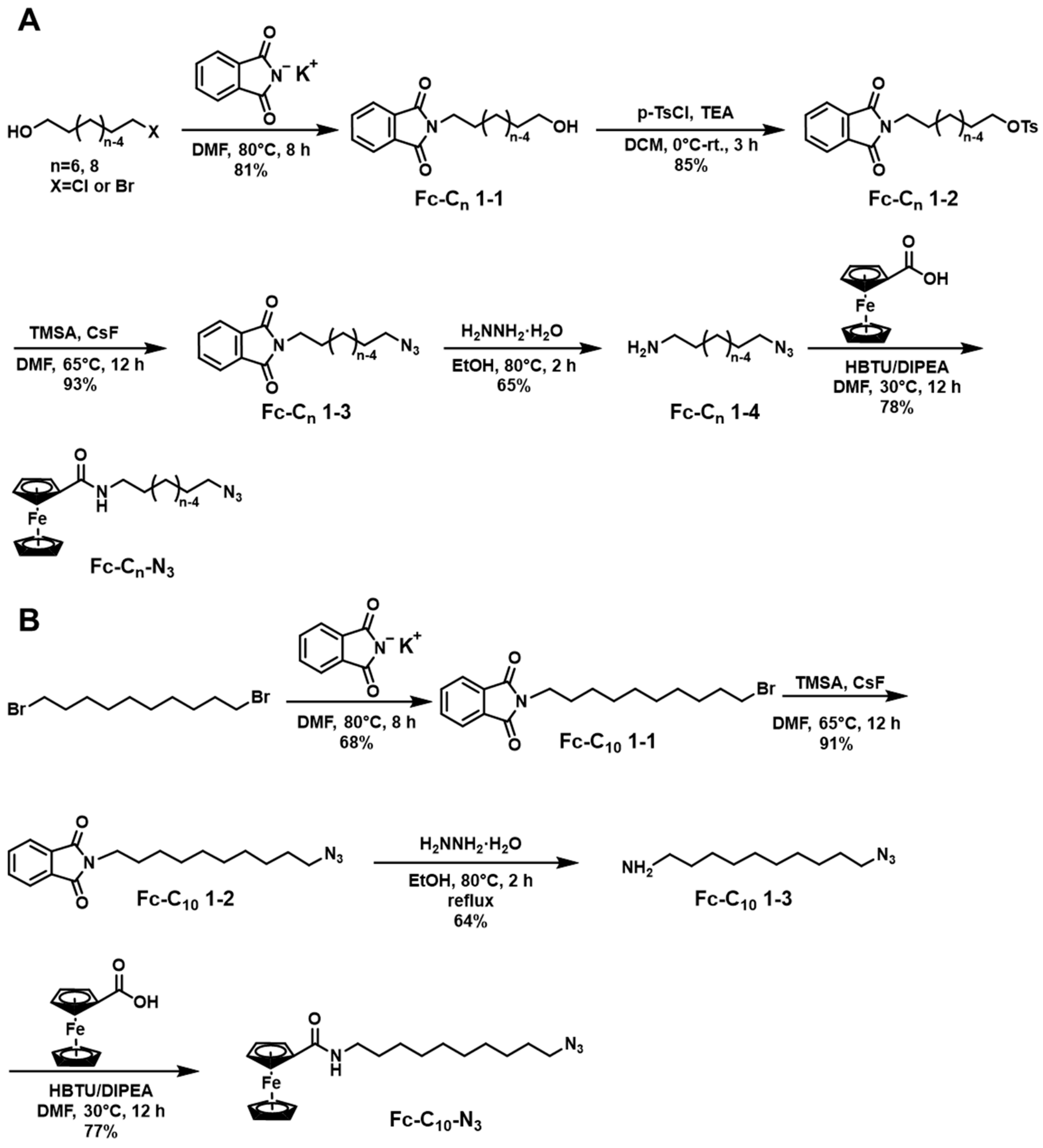

Figure S1. (A) ^{1}H NMR, (B) ^{13}CNMR, (C) HPLC, (D) ESI-HRMS and (E) IR spectra of **Fc-C$_6$-AmD 8A**.

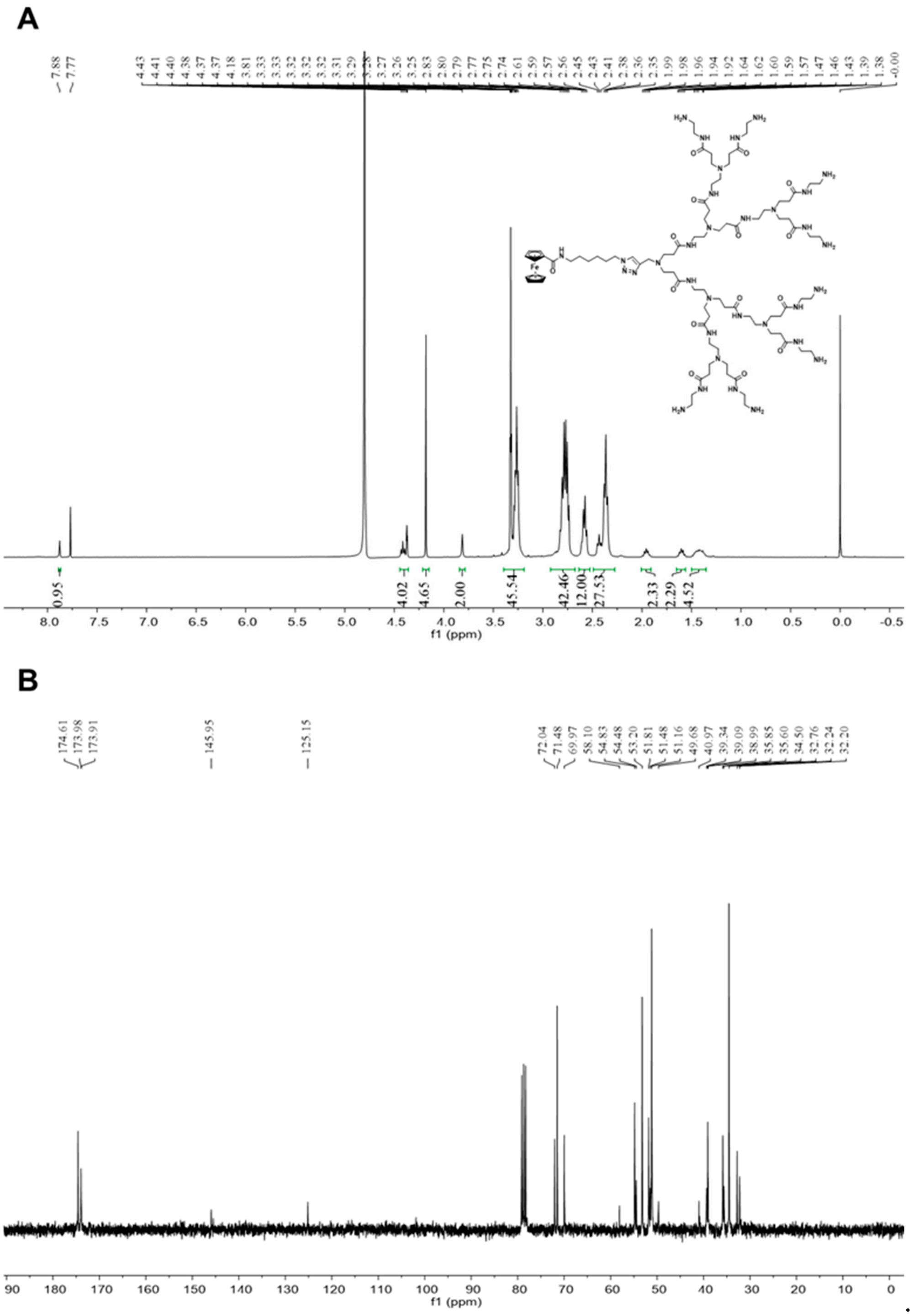
A
7.88
7.77
4.43
4.41
4.40
4.38
4.37
4.37
4.18
3.81
3.33
3.33
3.32
3.32
3.32
3.31
3.29
3.27
3.26
3.25
2.83
2.80
2.79
2.77
2.75
2.74
2.61
2.59
2.57
2.56
2.45
2.43
2.41
2.38
2.36
2.35
1.99
1.98
1.96
1.94
1.92
1.64
1.62
1.60
1.59
1.57
1.47
1.46
1.43
1.39
1.38
-0.00
0.95
4.02
4.65
2.00
45.54
42.46
12.00
27.53
2.33
2.29
4.52
f1 (ppm)
B
174.61
173.98
173.91
145.95
125.15
72.04
71.48
69.97
58.10
54.83
54.48
53.20
51.81
51.48
51.16
49.68
40.97
39.34
39.09
38.99
35.85
35.60
34.50
32.76
32.24
32.20
f1 (ppm)

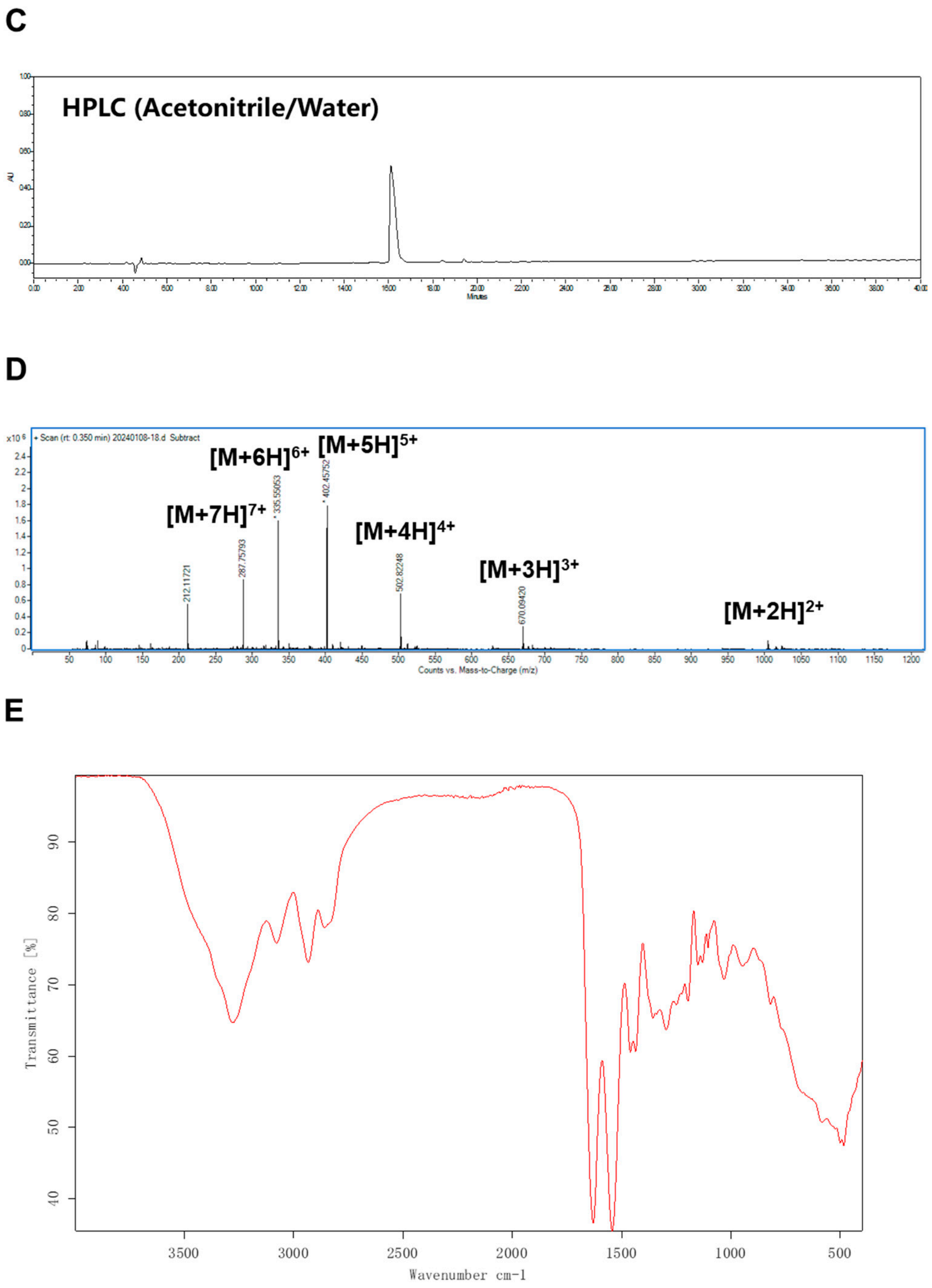

Figure S2. (A) ^{1}H NMR, (B) ^{13}CNMR, (C) HPLC, (D) ESI-HRMS and (E) IR spectra of **Fc-C$_8$-AmD 8A**.

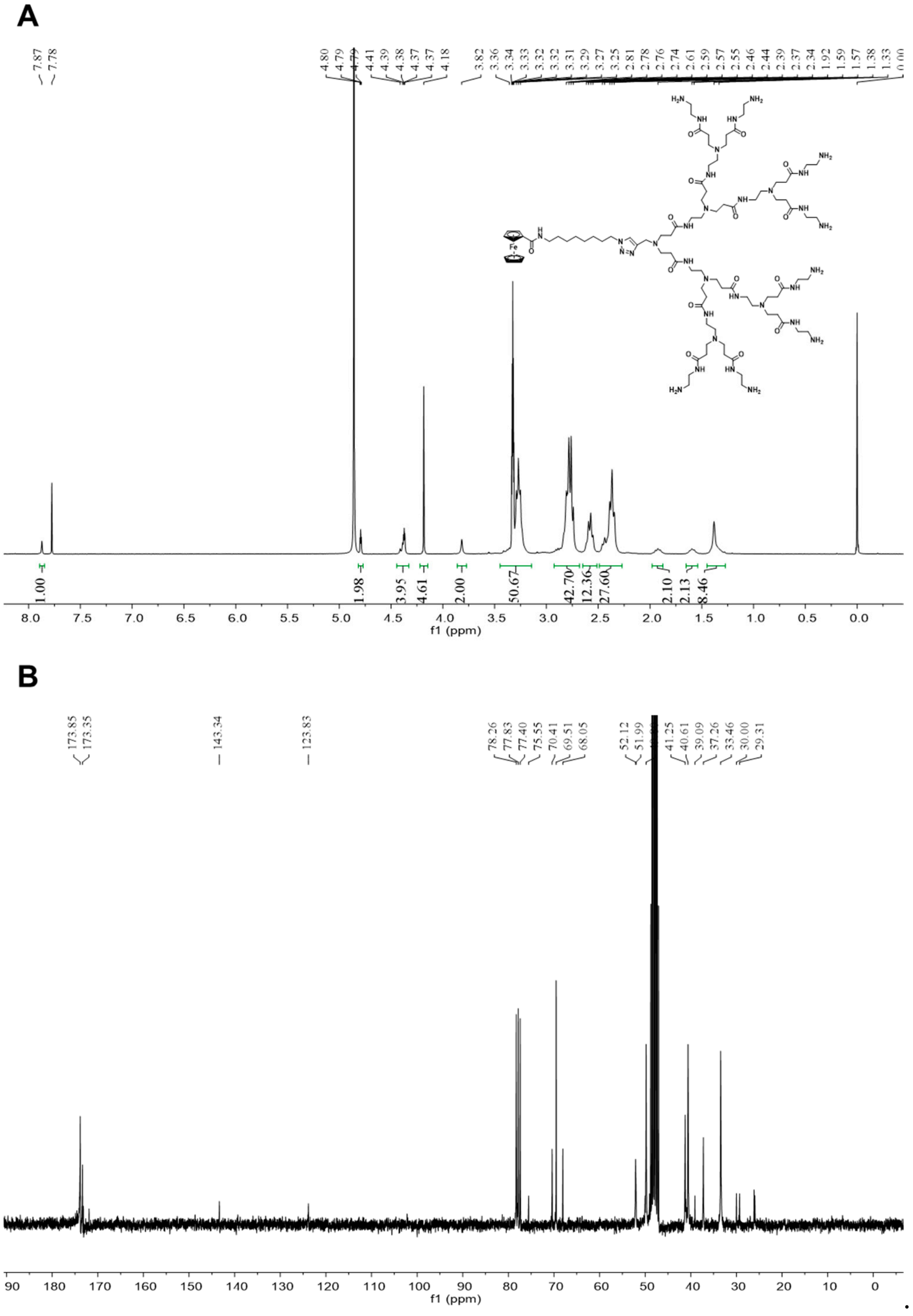
A
1.00
1.98
3.95
4.61
2.00
50.67
42.70
12.36
27.60
2.10
2.13
8.46
f1 (ppm)
B
f1 (ppm)

C

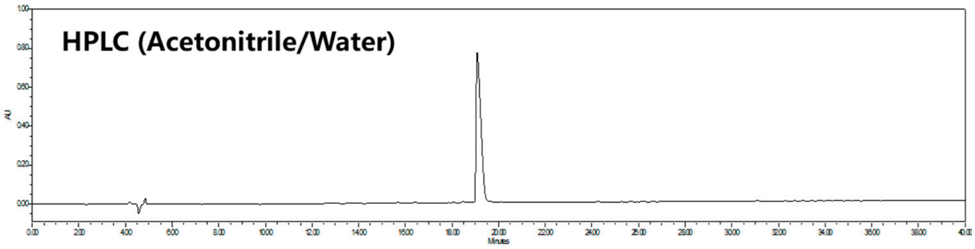
HPLC (Acetonitrile/Water)
AU
Minutes

D

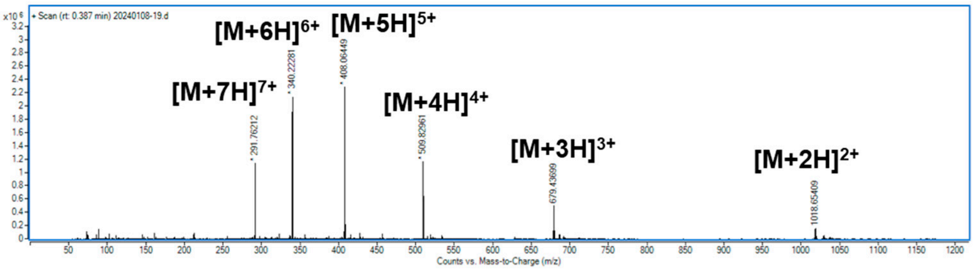
Scan (rt: 0.387 min) 20240108-19.d
[M+7H]7+
291.76212
[M+6H]6+
340.22281
[M+5H]5+
408.06449
[M+4H]4+
509.82961
[M+3H]3+
679.43699
[M+2H]2+
1018.65409
Counts vs. Mass-to-Charge (m/z)

E

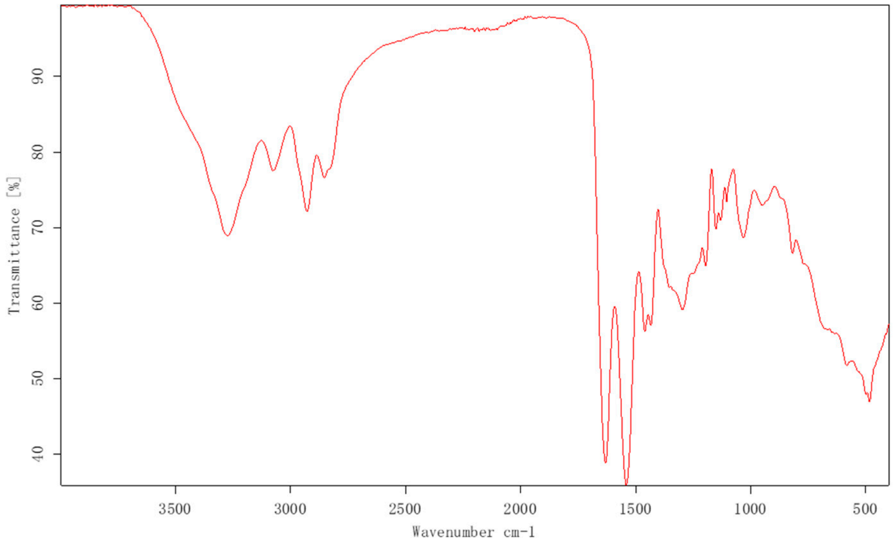
Transmittance [%]
Wavenumber cm-1
3500
3000
2500
2000
1500
1000
500

Figure S3. (A) ^{1}H NMR, (B) ^{13}CNMR, (C) HPLC, (D) ESI-HRMS and (E) IR spectra of **Fc-C$_{10}$-AmD 8A**.

A

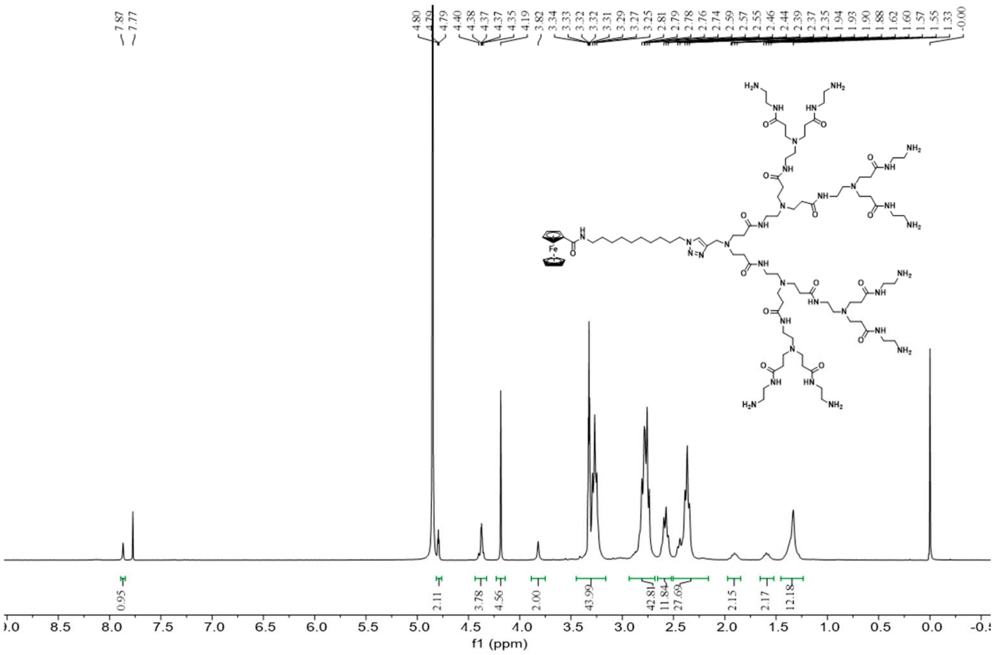

B

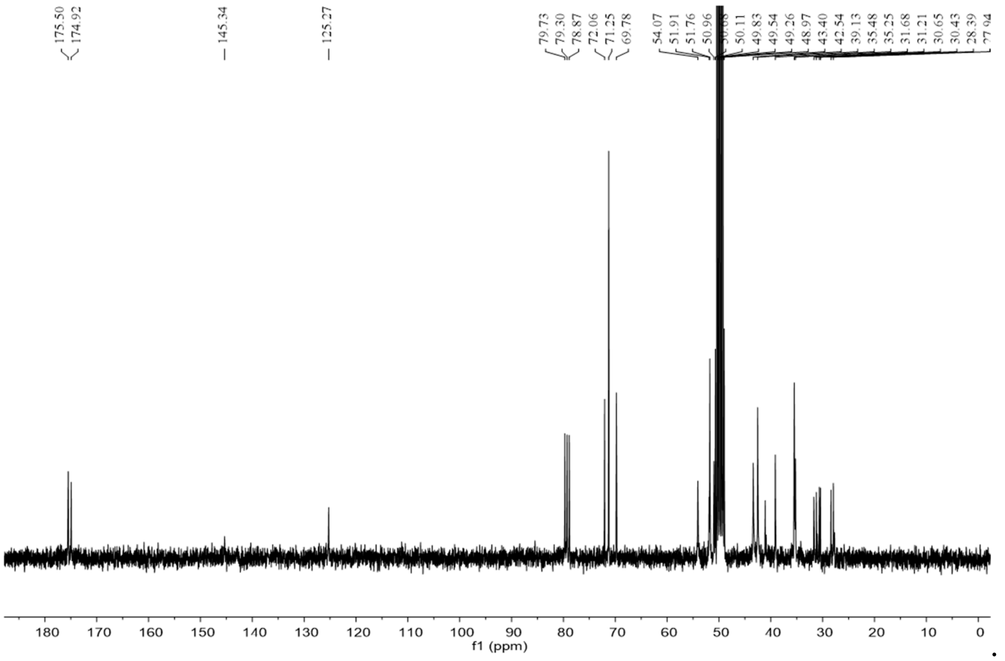

C

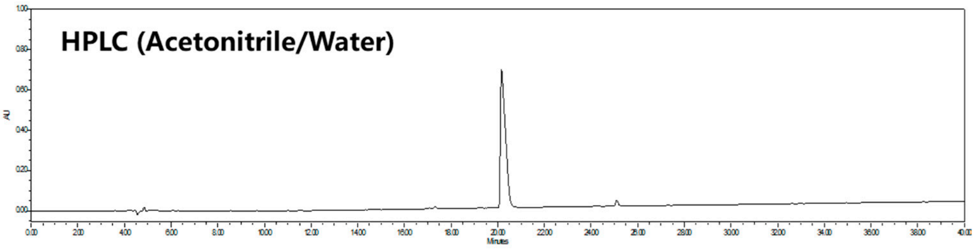
HPLC (Acetonitrile/Water)
AU
Minutes

D

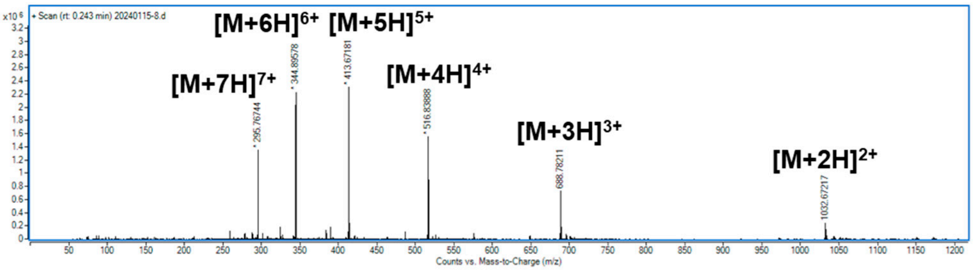
+ Scan (rt: 0.243 min) 20240115-8.d
[M+7H]7+
[M+6H]6+
[M+5H]5+
[M+4H]4+
[M+3H]3+
[M+2H]2+
295.76744
344.89578
413.67181
516.83888
688.78211
1032.67217
Counts vs. Mass-to-Charge (m/z)

E

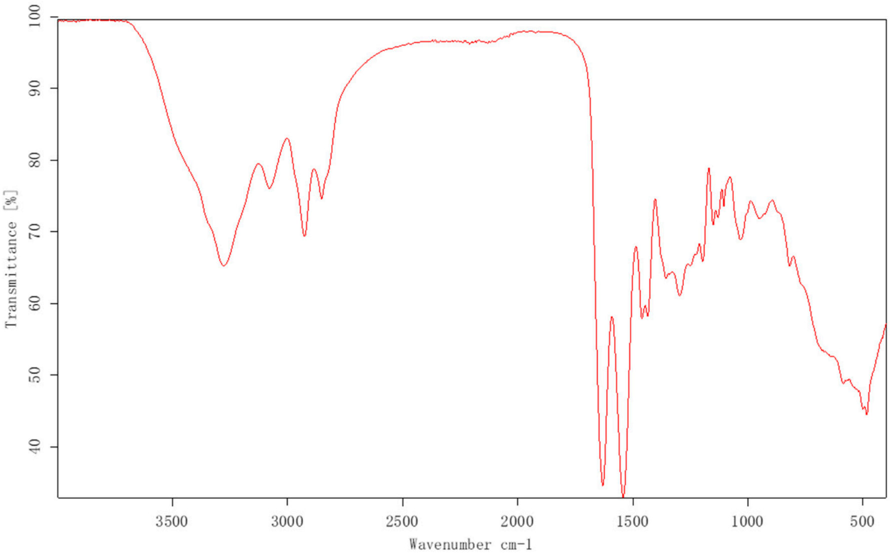
Transmittance [%]
Wavenumber cm-1

.

Figure S4. Potentiometric pH titration profile of **Fc-AmDs**.

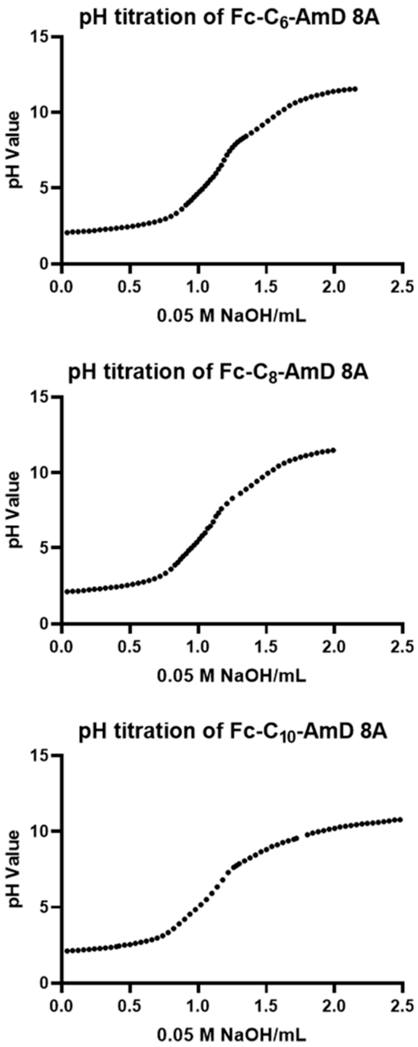

Figure S5. The ROS-responsive performance of **Fc-C8-AmD 8A**. The UV-vis absorption spectra of different groups (H_2O_2+TMB, **Fc-C8-AmD 8A** + TMB and **Fc-C8-AmD 8A** + TMB + H_2O_2) were measured under 200 mM H_2O_2.

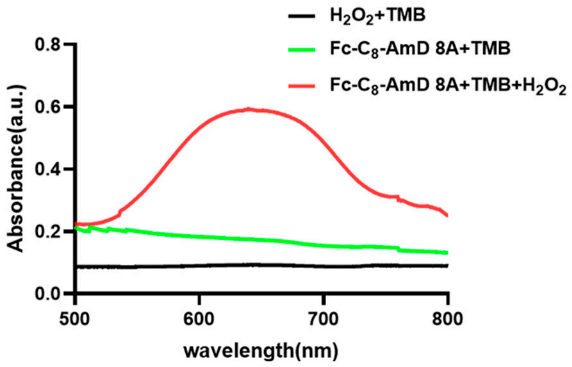

Figure S6. The CAC measurements of **Fc-AmDs** using pyrene fluorescent probe.

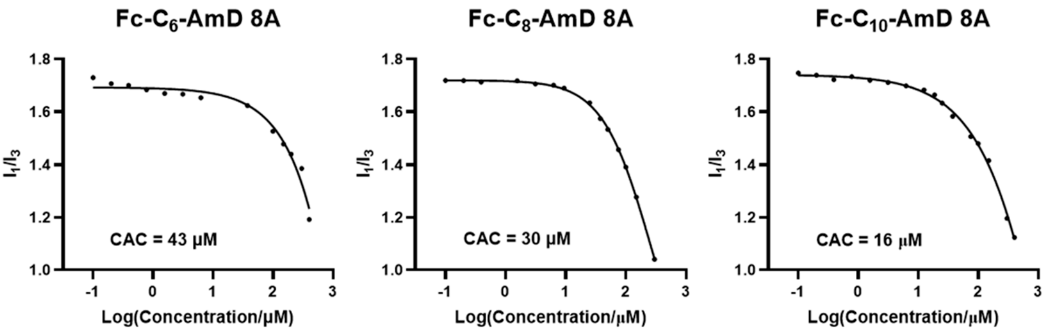

Figure S7. The MTT assay of the metabolic toxicity of **Fc-AmDs** in MDCK cells, L02 cells, L929 cells and SKOV-3 cells. (mean ± SD, *n* = 3).

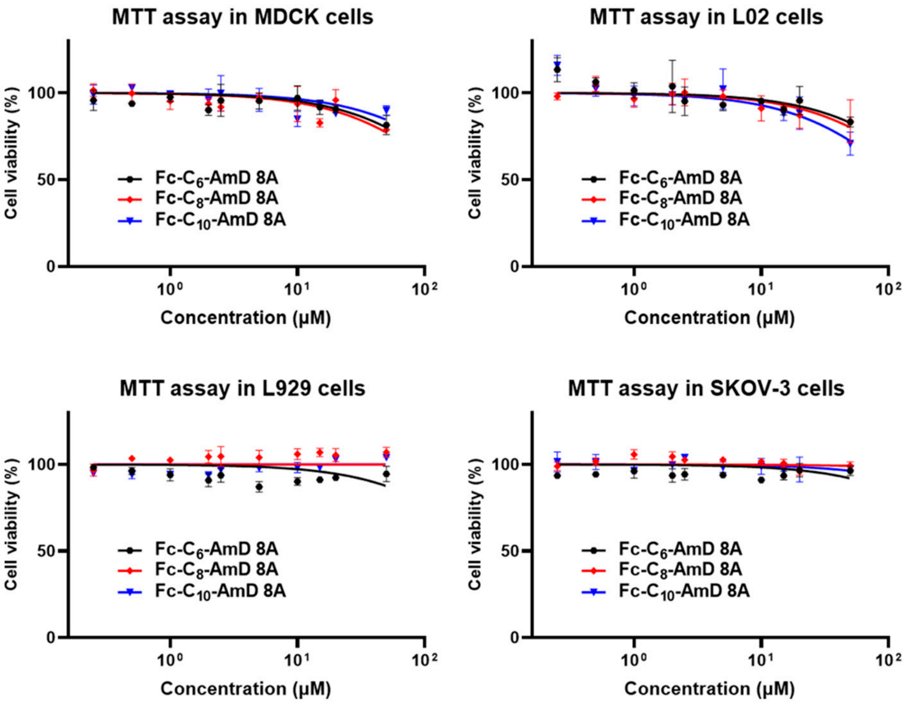

Figure S8. AKT2 protein expression on SKOV-3 cells after treatment with siRNA/**Fc-AmDs** complexes quantified by Western blotting (50 nM siRNA, N/P ratio of 10). *** $p \leq 0.001$ (mean ± SD, n = 3).

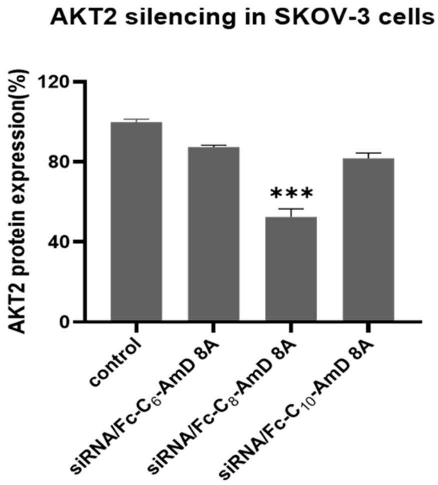

Figure S9. Size distribution of **Fc-C$_8$-AmD 8A** and siRNA/**Fc-C$_8$-AmD 8A** complexes by intensity determined using dynamic light scattering analysis. 200 μM **Fc-C$_8$-AmD 8A** was used for the DLS detection, whereas siRNA/**Fc-C$_8$-AmD 8A** complexes with N/P ratio of 10 and 1.0 μM siRNA was used for the DLS detection.

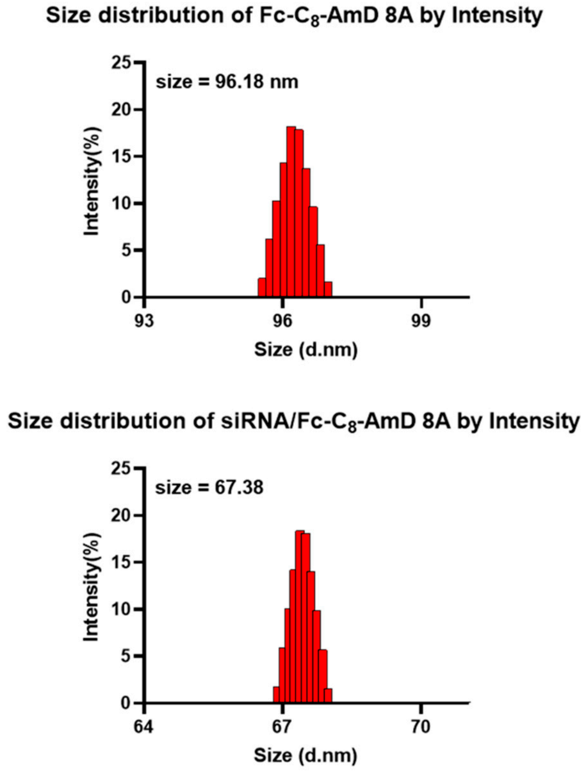

Figure S10 Agarose gel retardation of siRNA/Fc-C8-AmD 8A complexes at different time after incubation with RNase A and SDS (200 ng siRNA/well, N/P ratio of 10).

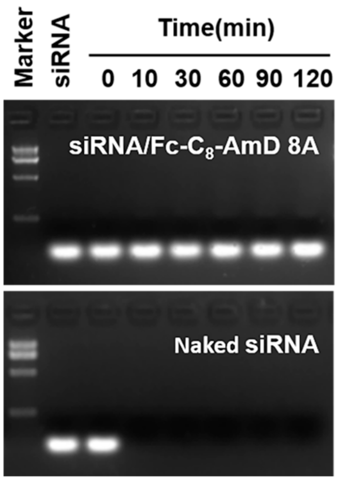

Figure S11. (A) Flow cytometry analysis and (B) confocal imaging of the cell uptake of the siRNA/**Fc-C8-AmD 8A** complexes in SKOV-3 cells (50 nM Cy5-labeled siRNA, N/P ratio of 10). Red: Cy5-labeled siRNA; Blue: Hoechst33342 labelling nuclei, Scale 20 μm.

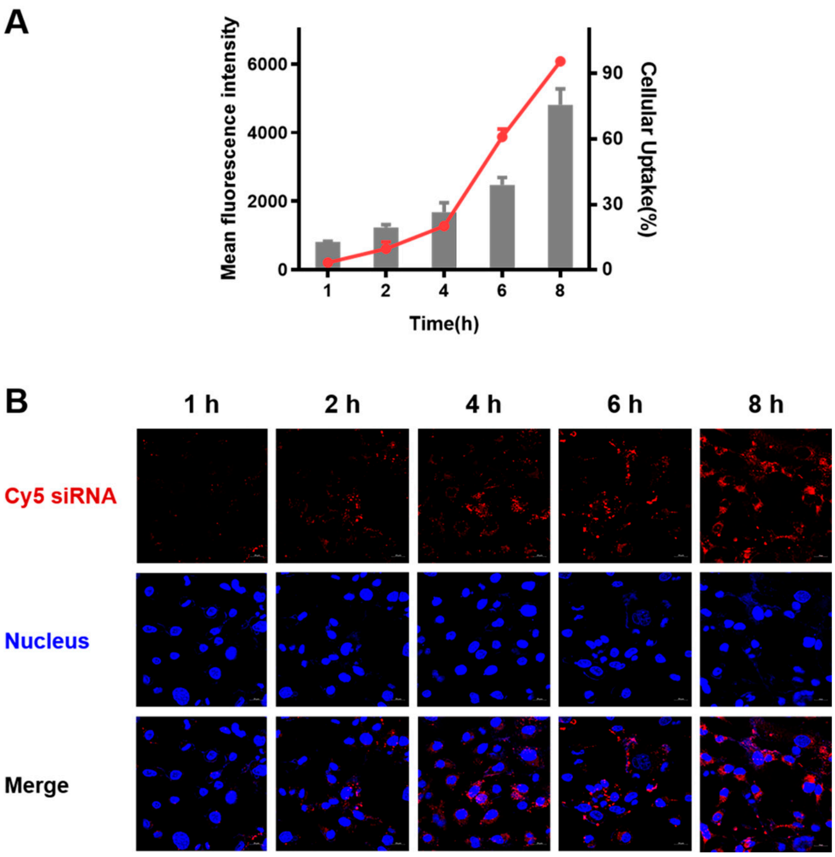

Figure S12. ROS-responsive siRNA delivery mediated by **Fc-C8-AmD 8A** in ROS-rich SKOV-3 cells and ROS-poor SKOV-3 cells (pretreated with antioxidant NAC) (50 nM siRNA, N/P ratio of 10). *** $p \leq 0.001$ (mean ± SD, $n = 3$).

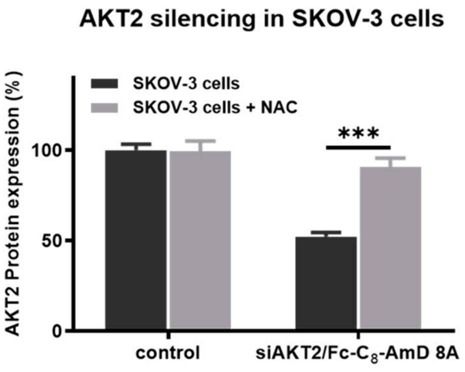

Figure S13. ROS levels in human ovarian cancer SKOV-3 cells, and SKOV-3 cells pretreated with the antioxidant N-acetyl-cysteine (NAC) (10 mM) quantified using CellROX orange reagent by flow cytometry. *** $p \leq 0.001$ (mean ± SD, $n = 3$).

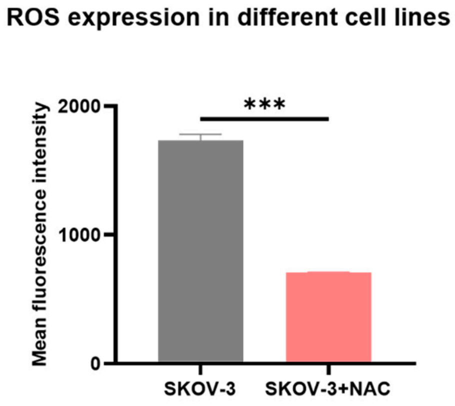

Figure S14. Gene silencing following siAKT2 delivery mediated by **8A**, **Fc-C8-N3** and **Fc-C8-AmD 8A** (50 nM siRNA, N/P ratio of 10). *** $p \leq 0.001$ (mean ± SD, $n = 3$).

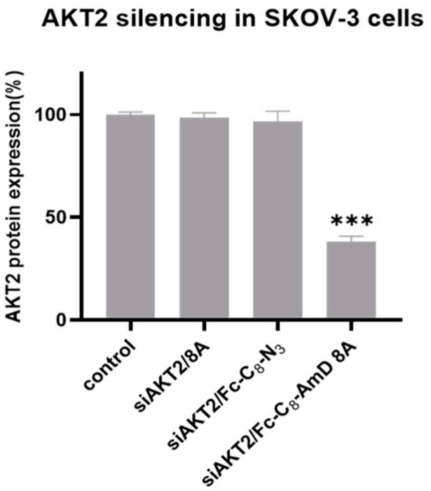

Figure S15. Dioleoylphosphatidylethanolamine (DOPE) enhanced the gene silencing of AKT2 after treatment of siRNA/**Fc-C8-AmD 8A** complexes with 50 nM siRNA at N/P ratio 10 on SKOV-3 cells. *** $p \leq 0.001$ (mean ± SD, $n = 3$).

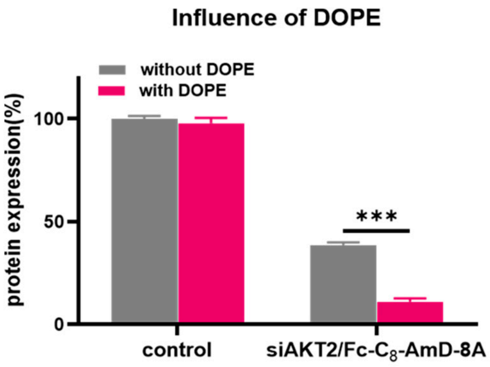

Figure S16. AKT2 protein downregulation following treatment with the siAKT2/**Fc-C8-AmD 8A** complex (50 nM siRNA, N/P ratio of 10) on SKOV-3 cells in the presence and absence of the proton pump inhibitor, bafilomycin A1. *** $p \leq 0.001$ (mean ± SD, $n = 3$).

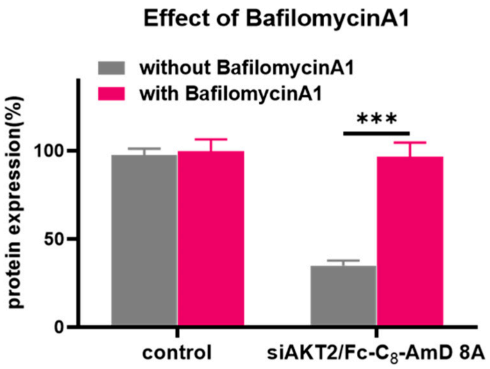

Table S1. The information of **Fc-AmDs** from HPLC detection.

Dendrimers	Retention Time (min)	Area (μV*sec)	Height (μV)	%Area
Fc-C6-AmD 8A	16.108	8852622	521292	98
Fc-C8-AmD 8A	19.082	10694744	771092	100
Fc-C10-AmD 8A	20.163	10188640	685108	98

Table S2. The IC_{50} of **Fc-AmDs** was assessed by MTT assay on normal and cancer cells.

**Dendrimers**	**IC_{50} of dendrimer on different cells/μM**			
	**L02 cells**	**L929 cells**	**MDCK cells**	**SKOV-3 cells**
Fc-C_6-AmD 8A	> 50	> 50	> 50	> 50
Fc-C_8-AmD 8A	> 50	> 50	> 50	> 50
Fc-C_{10}-AmD 8A	> 50	> 50	> 50	> 50

Synthesis and characterization of ferrocenyl amphiphilic dendrimers

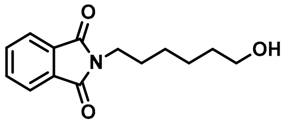

Fc-C_6 1-1: Take 6-chloro-1-hexanol (1381.2 mg, 10.11 mmol), potassium phthalimide (3816.9 mg, 20.61 mmol) in a reaction flask, and add DMF (25.0 mL) to dissolve the system. The system was reacted at 80℃ for 8 h under nitrogen. After removing DMF the reaction solution was diluted with water, extracted with EA. the organic phases were combined, washed with saturated sodium chloride solution, dried over anhydrous sodium sulfate, filtered and concentrated to obtain a white solid crude product. After purification by column chromatography (PE/EA = 2/1), **Fc-C_6 1-1** (2023.1 mg, 81%) was obtained as white solid. ^{1}H NMR (300 MHz, $CDCl_3$) δ 7.90 – 7.81 (m, 2H), 7.77 – 7.66 (m, 2H), 3.75 – 3.58 (m, 4H), 1.76 – 1.49 (m, 4H), 1.48 – 1.22 (m, 4H).

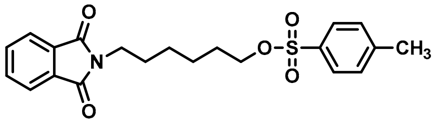

Fc-C_6 1-2: Fc-C_6 1-1 (1999.2 mg, 8.08 mmol), TEA (2.3 mL，16.5 mmol) and DCM (4.0 mL) were taken into a reaction flask, the solution of *p*-TsCl (3045.0 mg, 16.0 mmol) in DCM (20.0 mL) was added into the reaction flask with stirring in an ice bath. Then, the reaction was carried out at 30℃ for 3 h. After removing the reaction solution, the residue was diluted by adding water, extracted with EA. The organic phases were combined, washed with saturated sodium bicarbonate solution and saturated sodium chloride solution sequentially, dried with anhydrous sodium sulfate, filtered and concentrated to obtain a light yellow oil. After purification by column chromatography (PE/EA = 1/1), **Fc-C_6 1-2** (2793.1 mg, 89%) was obtained as colorless oil. ^{1}H NMR (300 MHz, $CDCl_3$) 7.88 – 7.66 (m, 6H), 7.34 (d, *J* = 8.1 Hz, 2H), 4.01 (t, *J* = 6.4 Hz, 2H), 3.63 (t, *J* = 7.2 Hz, 2H), 2.44 (s, 3H), 1.72 – 1.51 (m, 4H), 1.43 – 1.20 (m, 4H).

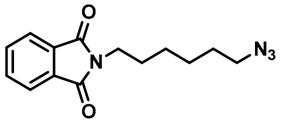

Fc-C_6 1-3: Take **Fc-C_6 1-2** (2781.8 mg, 7.18 mmol), CsF (2807.4 mg, 18.5 mmol) and DMF (15.0 mL) into a reaction flask under argon, and then TMSA (2.4 mL, 18.0 mmol) was added dropwise with stirring at room temperature, the reaction was carried out at 65°C for 12 h under dark. After removing DMF under reduced pressure, the residue was diluted with water, extracted with EA, the organic phases were combined, washed with saturated sodium chloride solution, dried over anhydrous sodium sulfate, and filtered and concentrated to obtain a light yellow oil. After purification by column chromatography (PE/EA = 4/1), **Fc-C_6 1-3** (1823.4 mg, 93%) was obtained as a colourless oil. ^{1}H NMR (300 MHz, $CDCl_3$) δ 7.90 – 7.80 (m, 2H), 7.76 – 7.67 (m, 2H), 3.69 (t, *J* = 6.0 Hz, 2H), 3.26 (t, *J* = 6.8 Hz, 2H), 1.77 – 1.51 (m, 4H), 1.50 – 1.31 (m, 4H). IR (cm^{-1}): *υ* 2090.69 (-N_3).

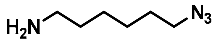

Fc-C$_6$ 1-4: Fc-C$_6$ 1-3 (1045.1 mg, 3.84 mmol) and EtOH (25.0 mL) were taken into a reaction flask, hydrazine hydrate (1.4 mL, 28.9 mmol) was added while stirring at room temperature, and the reaction was stirred at 80°C for 2 h. At the end of the reaction, the reaction solution was filtered and the filtrate was concentrated. The crude product was diluted with water, extracted with DCM. The DCM phase was combined, washed with saturated sodium chloride solution, dried over anhydrous sodium sulfate, filtered and concentrated to give **Fc-C$_6$ 1-4** (398.1 mg, 73%) as a colourless oil. ^{1}H NMR (300 MHz, CDCl$_3$) δ 3.27 (t, *J* = 6.9 Hz, 2H), 2.70 (t, *J* = 6.8 Hz, 2H), 1.72 – 1.23 (m, 8H). IR (cm^{-1}): υ 2091.37 (-N$_3$).

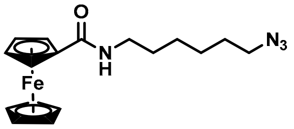

Fc-C$_6$-N$_3$: Ferrocenecarboxylic acid (773.3 mg, 3.36 mmol), HBTU (1276.3 mg, 3.37 mmol) and DMF (15.0 mL) were taken into a reaction flask under argon, and DIPEA (2.4 mL, 13.8 mmol) was added slowly with stirring in an ice bath, stirring for 1 h. After returning to room temperature, DMF solution (7 mL) of **Fc-C$_6$ 1-4** (398.1 mg, 2.80 mmol) was slowly added to the above reaction solution and the reaction was stirred at 30°C for 12 h. After removing the DMF under reduced pressure, the residue was diluted with water, extracted with DCM, the organic phases were combined, washed with saturated sodium bicarbonate solution and saturated sodium chloride solution sequentially, dried over anhydrous sodium sulfate, and filtered and concentrated to obtain a yellow oil. After purification by column chromatography (PE/EA = 2/1), **Fc-C$_6$-N$_3$** (764.8 mg, 78%) was obtained as a yellow solid. ^{1}H NMR (300 MHz, CDCl$_3$) δ 5.69 (br, 1H), 4.65 (t, *J* = 1.9 Hz, 2H), 4.34 (t, *J* = 1.7 Hz, 2H), 4.20 (s, 5H), 3.38 (q, *J* = 6.7 Hz, 2H), 3.28 (t, *J* = 6.8 Hz, 2H), 1.71 – 1.54 (m, 4H), 1.52 – 1.34 (m, 4H). ^{13}C NMR (75 MHz, CDCl$_3$) δ 170.20, 70.36, 69.72, 68.06, 51.37, 39.41, 29.92, 28.78, 26.52, 26.47. ESI-MS(*m*/*z*): calcd for $C_{17}H_{22}FeN_4O$, $[M+H]^+$ 355.1, found 355.1. IR (cm^{-1}): υ 2087.22 (-N$_3$).

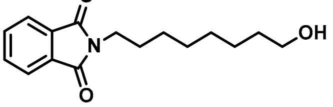

Fc-C$_8$ 1-1: 8-Bromo-1-octanol (829.4 mg, 3.97 mmol) and potassium phthalimide (1105.2 mg, 5.97 mmol) were taken into a reaction flask, and the synthesis process was consistent with that of **Fc-C$_6$ 1-1**, resulting in a white solid **Fc-C$_8$ 1-1** (981.3 mg, 90%). ^{1}H NMR (300 MHz, CDCl$_3$) δ 7.91 – 7.79 (m, 2H), 7.77 – 7.65 (m, 2H), 3.75 – 3.58 (m, 4H), 1.77 – 1.44 (m, 4H), 1.42 – 1.24 (m, 8H).

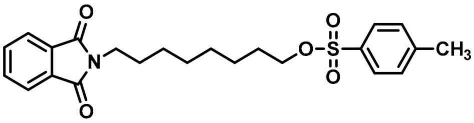

Fc-C$_8$ 1-2: Fc-C$_8$ 1-1 (1349.0 mg, 4.90 mmol) was taken in a reaction flask and the synthesis was carried out in the same manner as for **Fc-C$_6$ 1-2**, resulting in a white solid **Fc-C$_8$ 1-2** (1780.9 mg, 85%). ^{1}H NMR (300 MHz, CDCl$_3$) δ 7.89 – 7.67 (m, 6H), 7.35 (d, *J* = 9.0 Hz, 2H), 4.00 (t, *J* = 6.5 Hz, 2H), 3.66 (t, *J* = 6.0 Hz, 2H), 2.45 (s, 3H), 1.72 – 1.51 (m, 4H), 1.38 – 1.14 (m, 8H).

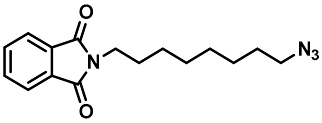

Fc-C$_8$ 1-3: Fc-C$_8$ 1-2 (1311.0 mg, 3.05 mmol) and CsF (1171.1 mg, 7.71 mmol) were taken in a reaction flask, and the synthesis was carried out in the same way as for **Fc-C$_6$ 1-3**, resulting in a white solid **Fc-C$_8$ 1-3** (884.6 mg, 95%). ^{1}H NMR (300 MHz, CDCl$_3$) δ 7.89 – 7.80 (m, 2H), 7.76 – 7.67 (m, 2H), 3.68 (t, *J* = 7.5 Hz, 2H), 3.25 (t, *J* = 7.0 Hz, 2H), 1.77 – 1.50 (m, 4H), 1.43 – 1.23 (m, 8H).

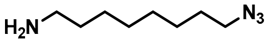

Fc-C8 1-4: Fc-C8 1-3 (943.6 mg, 3.14 mmol) was taken in a reaction flask, and the rest of the procedure was referred to the synthesis of **Fc-C6 1-4**. A colourless oily liquid **Fc-C8 1-4** (372.8 mg, 65 %) was obtained. ^{1}H NMR (400 MHz, $CDCl_3$) δ 3.26 (t, *J* = 7.0 Hz, 2H), 2.68 (t, *J* = 7.0 Hz, 2H), 1.66 – 1.54 (m, 2H), 1.51 – 1.23 (m, 10H). IR (cm^{-1}): *υ* 2089.69 ($-N_3$).

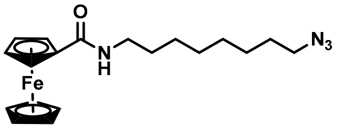

Fc-C8-N3: Ferrocenecarboxylic acid (414.5 mg, 1.80 mmol), HBTU (683.3 mg, 1.80 mmol) were taken in a reaction flask and synthesized as for **Fc-C6-N3**. A yellow solid **Fc-C8-N3** (493.1 mg, 86%) was obtained. ^{1}H NMR (300 MHz, $CDCl_3$) δ 5.67 (br, 1H), 4.65 (t, *J* = 1.9 Hz, 2H), 4.33 (t, *J* = 1.6 Hz, 2H), 4.20 (s, 5H), 3.37 (q, *J* = 6.6 Hz, 2H), 3.26 (t, *J* = 6.9 Hz, 2H), 1.69 – 1.51 (m, 4H), 1.47 – 1.28 (m, 8H). ^{13}C NMR (75 MHz, $CDCl_3$) δ 170.12, 70.33, 69.72, 68.03, 51.45, 39.54, 29.99, 29.20, 29.09, 28.82, 26.88, 26.66. ESI-MS(*m*/*z*): calcd for $C_{19}H_{26}FeN_4O$, $[M+H]^+$ 383.2, found 383.2. IR (cm^{-1}): *υ* 2091.63 ($-N_3$).

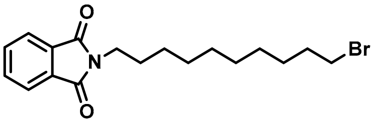

Fc-C10 1-1: 1, 10-dibromodecane (4508.0 mg, 15.0 mmol) and DMF (20 mL) were taken into a reaction flask, and the solution of potassium phthalimide (922.1 mg, 4.98 mmol) in DMF (10.0 mL) was slowly added dropwise with stirring under an ice bath, and after the system was brought back to room temperature, the reaction was carried out at 80°C for 8 h. The post-processing procedure was consistent with that of **Fc-C6 1-1**. A white solid **Fc-C10 1-1** (1245.1 mg, 68%) was obtained by purification on column chromatography (PE/EA = 10/1). ^{1}H NMR (300 MHz, $CDCl_3$) δ 7.88 – 7.81 (m, 2H), 7.76 – 7.68 (m, 2H), 3.68 (t, *J* = 7.5 Hz, 2H), 3.40 (t, *J* = 6.9 Hz, 2H), 1.84 (p, *J* = 6.9 Hz, 2H), 1.74 – 1.61 (m, 2H), 1.47 – 1.20 (m, 12H). ^{13}C NMR (75 MHz, $CDCl_3$) δ 168.36, 134.01, 132.03, 123.26, 36.95, 32.84, 29.84, 27.24.

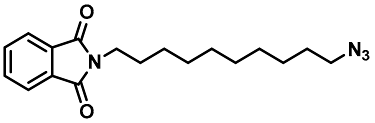

Fc-C10 1-2: Fc- C10 1-1 (1397.1 mg, 3.81 mmol) was taken in a reaction flask and the post-processing was carried out as for **Fc-C6 1-3**, resulting in a white solid, **Fc-C10 1-2** (1139.0 mg, 91%) by purification on column chromatography (PE/EA = 10/1). ^{1}H NMR (300 MHz, $CDCl_3$) δ 7.89 – 7.80 (m, 2H), 7.75 – 7.67 (m, 2H), 3.68 (t, *J* = 7.5 Hz, 2H), 3.25 (t, *J* = 6.9 Hz, 2H), 1.75 – 1.52 (m, 4H), 1.42 – 1.21 (m, 12H).

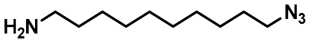

Fc-C10 1-3: Fc-C10 1-2 (943.5 mg, 2.87 mmol) was taken in a reaction flask and the rest of the procedure was referred to the synthesis of **Fc-C6 1-4**. A colorless oil **Fc-C10 1-3** (363.4 mg, 64%) was obtained. ^{1}H NMR (300 MHz, $CDCl_3$) δ 3.26 (t, *J* = 6.9 Hz, 2H), 2.69 (t, *J* = 6.9 Hz, 2H), 1.71 – 1.14 (m, 16H). ^{13}C NMR (75 MHz, $CDCl_3$) δ 51.35, 42.04, 33.58, 29.32, 29.28, 29.25, 28.96, 28.68, 26.71, 26.55. IR (cm^{-1}): *υ* 2091.63 ($-N_3$).

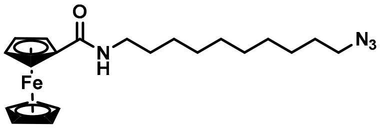

Fc-C10-N3: Fc-C10 1-3 (358.9 mg, 1.81 mmol) was taken in a reaction flask and the rest of the procedure was referred to the synthesis of **Fc-C6-N3**. A yellow solid **Fc-C10-N3** (606.5 mg, 77%) was obtained by purification on column chromatography (PE/EA = 3/1). ^{1}H NMR (300 MHz, $CDCl_3$) δ 5.66 (br, 1H), 4.66 (br, 2H), 4.34 (br, 2H), 4.21 (s, 5H), 3.44 – 3.31 (m, 2H), 3.25 (t, *J* = 6.9 Hz, 2H), 1.67 – 1.52 (m, 4H), 1.46 – 1.25 (m, 12H). ^{13}C NMR (75 MHz, $CDCl_3$) δ 170.09, 70.30, 69.71, 68.05, 51.48, 39.58, 30.01, 29.46, 29.41, 29.32, 29.11, 28.83, 26.99,

26.70. ESI-MS(*m*/*z*): calcd for $C_{21}H_{30}FeN_4O$, $[M+H]^+$ 411.2, found 411.2. IR (cm^{-1}): υ 2091.04 ($-N_3$).

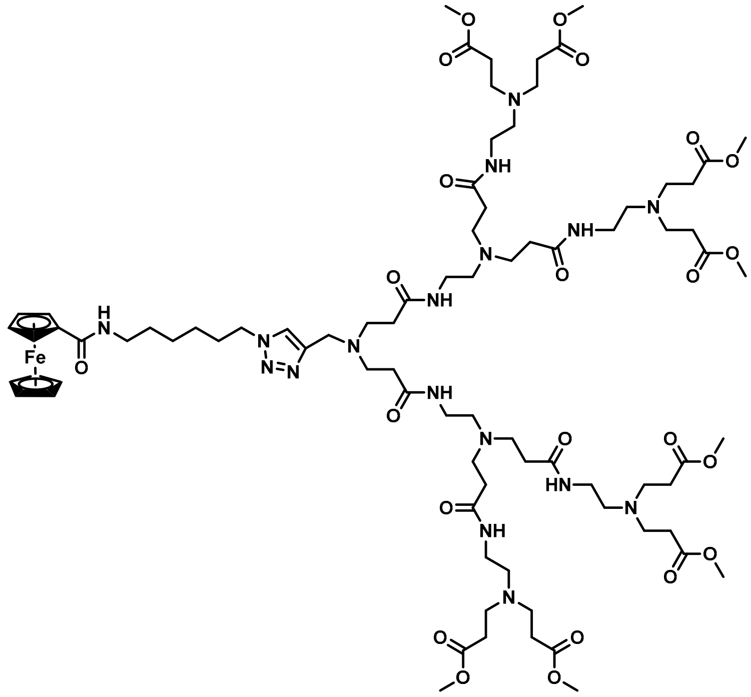

Fc-C$_6$-AmD 8E: Fc-C$_6$-N$_3$ (39.1 mg, 0.11 mmol), **8E** (140.3 mg, 0.10 mmol), CuI (10.2 mg, 0.07 mmol) and DMF (4.0 mL) were taken into a reaction flask, protected by argon. And DBU (0.12 mL, 0.80 mmol) was added at room temperature while stirring. The reaction was carried out at 50°C for 3 h under dark. After removing DMF under reduced pressure, the reaction solution was diluted with saturated NH_4Cl solution, extracted with DCM. The organic phases were combined, washed with saturated ammonium chloride solution and saturated sodium chloride solution sequentially, dried over anhydrous sodium sulfate, and filtered and concentrated to obtain the crude product. The crude product was purified by column chromatography (DCM/MeOH = 5/1), and obtained as yellow oil **Fc-C$_6$-AmD 8E** (127.8 mg, 72%). ^{1}H NMR (300 MHz, CD_3OD) δ 7.92 (s, 1H), 4.79 (br, 2H), 4.49 – 4.34 (m, 4H), 4.18 (s, 5H), 3.82 (s, 2H), 3.66 (s, 24H), 3.45 – 3.15 (m, 14H), 3.03 – 2.20 (m, 68H), 2.02 – 1.87 (m, 2H), 1.68 – 1.53 (m, 2H), 1.53 – 1.31(m, 4H). ESI-MS(*m*/*z*): calcd for $C_{82}H_{135}FeN_{17}O_{23}$, $[M+H]^+$ 1782.94, $[M+Na]^+$ 1805.9, found 1783.1, 1805.1. IR (cm^{-1}): υ 1731.07 (-COOC-).

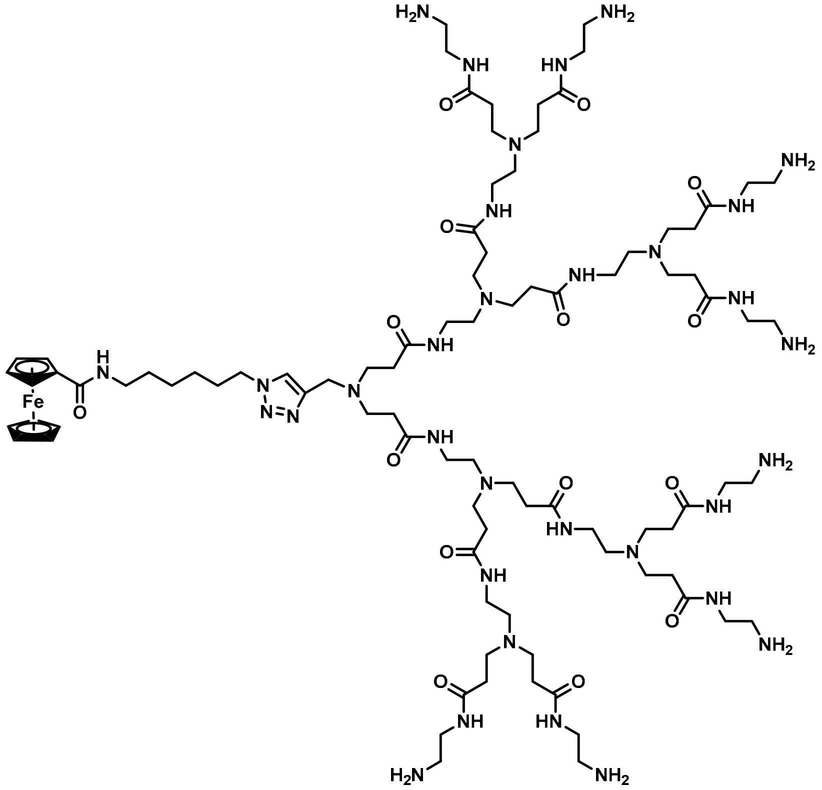

Fc-C6-AmD 8A: Fc-C6-AmD 8E (72.5 mg, 0.04 mmol) and methanol (2.4 mL) were taken into a reaction flask and protected by argon. And ethylenediamine (2.4 mL, 30.49 mmol) was added slowly by dropping at -20°C while stirring, after the reaction system was returned to room temperature, the reaction was carried out at 30°C under dark for 48 h and monitored by IR. The vibration band of carbonyl from the ester terminal of **Fc-C6-AmD 8E** dendrimer ($\upsilon_{C=O}$) at 1731 cm^{-1} disappearing, indicating that the amidation reaction was completed. After removing the solvent under reduced pressure, the crude product was obtained by precipitation with methanol/ether. The crude product was dissolved in deionized water, purified by dialysis, and lyophilized to give **Fc-C6-AmD 8A** (67.2 mg, 81%) as a yellow solid. ^1H NMR (300 MHz, $CD_3OD/CDCl_3$) δ 7.89 (s, 1H), 4.79 (br, 2H), 4.47 – 4.34 (m, 4H), 4.18 (s, 5H), 3.81 (s, 2H), 3.50 – 3.15 (m, 30H), 2.96 – 2.67 (m, 44H), 2.66 – 2.50 (m, 12H), 2.49 – 2.25 (m, 28H), 2.05 – 1.89 (m, 2H), 1.67 – 1.55 (m, 2H), 1.52-1.34 (m, 4H). ^{13}C NMR (75 MHz, $CD_3OD/CDCl_3$) δ 174.04, 173.36, 173.18, 171.93, 143.24, 123.85, 75.58, 70.43, 69.54, 68.05, 52.12, 49.81, 49.25,47.87, 40.39, 39.26, 37.26, 33.47, 30.05, 29.48, 29.01, 28.79, 26.70, 26.23. ESI-HRMS(*m*/*z*): calcd for $C_{90}H_{167}FeN_{33}O_{15}$, $[M+2H]^{2+}$ 1004.6424, found 1004.6393. HPLC (RT = 16.1 min). IR (cm^{-1}): υ 1630.50 and 1542.97 (-NH(CO)-).

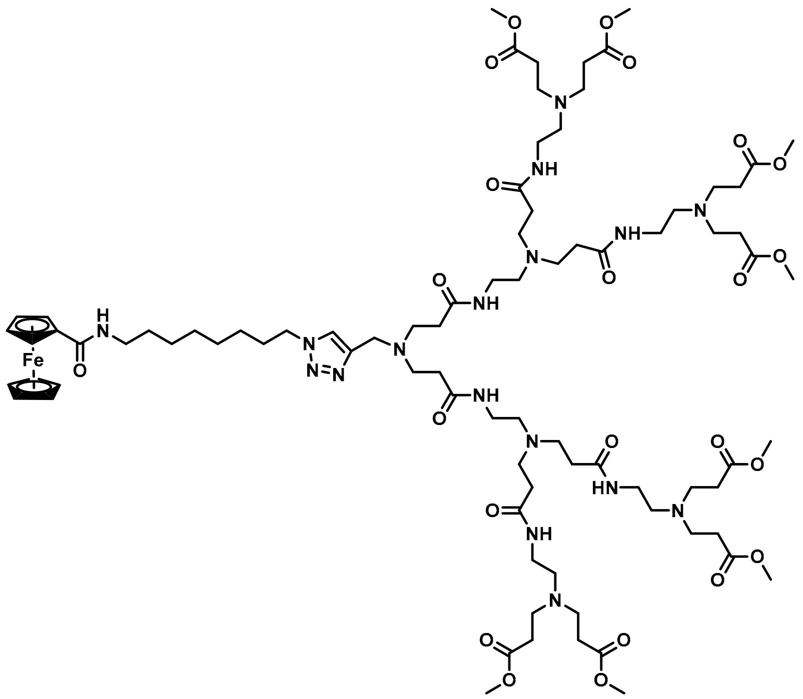

Fc-C$_8$-AmD 8E: Fc-C$_8$-N$_3$ (38.3 mg, 0.10 mmol) and **8E** (136.3 mg, 0.09 mmol) were taken into a reaction flask, and the rest of the operation in the synthesis was consistent with **Fc-C$_6$-AmD 8E**. A yellow oil **Fc-C$_8$-AmD 8E** (118.8 mg, 66%) was obtained. ^{1}H NMR (300 MHz, CDCl$_3$) δ 7.79 (br, 2H), 7.61 (s, 1H), 7.10 (br, 4H), 6.02 (br, 1H), 4.70 (br, 2H), 4.37 – 4.29 (m, 4H), 4.19 (s, 5H), 3.84 (s, 2H), 3.67 (s, 24H), 3.41 – 3.21 (m, 14H), 2.96 – 2.70 (m, 28H), 2.67 – 2.26 (m, 40H), 1.98 – 1.75 (m, 2H), 1.63 – 1.50 (m, 2H), 1.42 – 1.23 (m, 8H). ESI-MS(*m*/*z*): calcd for $C_{84}H_{139}FeN_{17}O_{23}$, $[M+H]^+$ 1811.0, $[M+Na]^+$ 1833.0, found 1811.3, 1833.3. IR (cm^{-1}): *υ* 1731.92 (-COOC-).

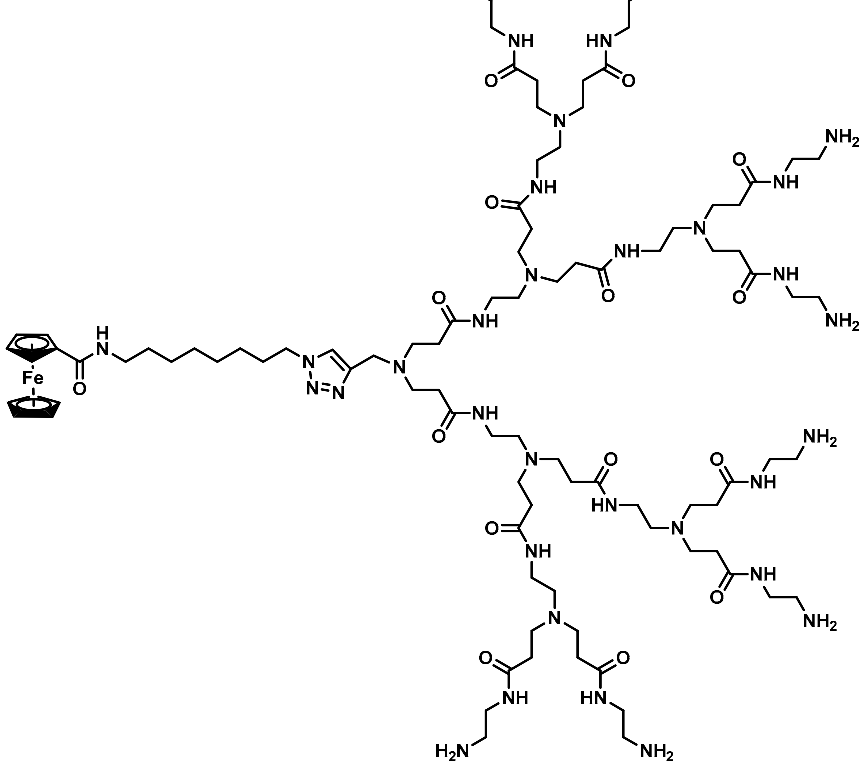

Fc-C$_8$-AmD 8A: Fc-C$_8$-AmD 8E (119.8 mg, 0.07 mmol) and methanol (1.5 mL) were taken into a reaction flask and protected by argon. And ethylenediamine (3.3 mL, 49.61 mmol) was added dropwise with stirring at -20°C. After the addition of ethylenediamine,

the reaction system was brought back to room temperature, and then the reaction was carried out at 30°C under dark. The rest of the synthesis was carried out according to **Fc-C$_6$-AmD 8A**, and **Fc-C$_8$-AmD 8A** (134.3 mg, 79%) was obtained as a yellow solid. ^{1}H NMR (300 MHz, $CD_3OD/CDCl_3$) δ 7.87 (s, 1H), 4.79 (br, 2H), 4.46 – 4.33 (m, 4H), 4.18 (s, 5H), 3.82 (s, 2H), 3.47 – 3.14 (m, 30H), 2.98 – 2.69 (m, 44H), 2.68-2.53 (m, 12H), 2.49 – 2.28 (m, 28H), 1.98 – 1.86 (m, 2H), 1.67 – 1.52 (m, 2H), 1.45 – 1.27 (m, 8H). ^{13}C NMR (75 MHz, $CD_3OD/CDCl_3$) δ 173.86, 173.36, 173.17, 171.93, 143.35, 123.84, 75.56, 70.42, 69.52, 68.05, 52.12, 49.80, 49.05, 47.68, 41.26, 40.61, 39.10, 37.26, 33.46, 30.00, 29.31, 26.13, 25.97. ESI-HRMS(*m*/*z*): calcd for $C_{92}H_{171}FeN_{33}O_{15}$, $[M+2H]^{2+}$ 1018.6581, found 1018.6541. HPLC (RT = 19.1 min). IR (cm^{-1}): *υ* 1631.17 and 1541.60 (-NH(CO)-).

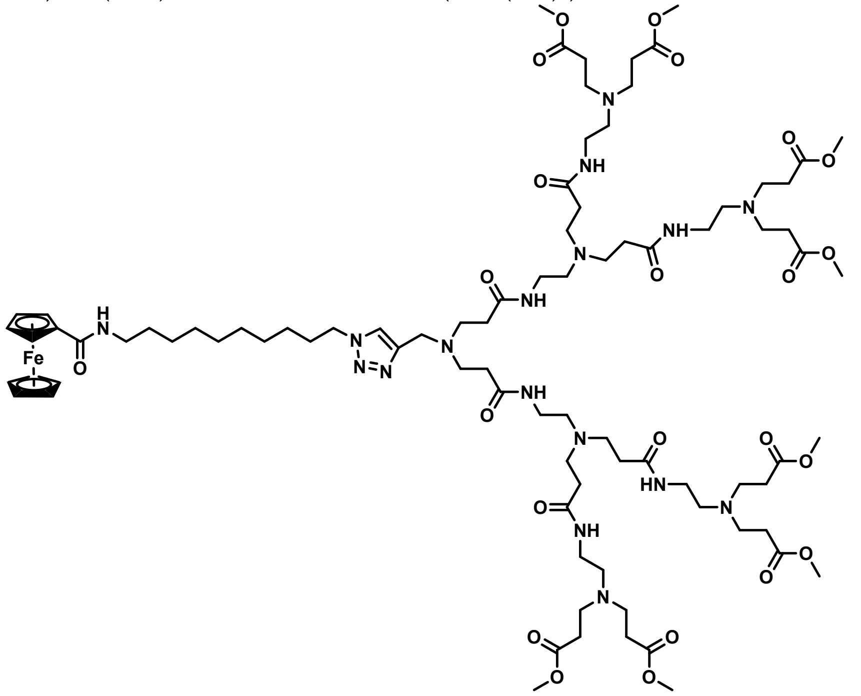

Fc-C$_{10}$-AmD 8E: **Fc-C$_{10}$-N$_3$** (45.6 mg, 0.11 mmol) and **8E** (140.8 mg, 0.10 mmol) were taken into a reaction flask. And the rest of the operation in the synthesis was consistent with that of **Fc-C$_6$-AmD 8E**. A yellow oil **Fc-C$_{10}$-AmD 8E** (126.5 mg, 69%) was obtained. ^{1}H NMR (300 MHz, $CDCl_3$) δ 7.78 (br, 2H), 7.61 (s, 1H), 7.10 (br, 4H), 5.90 (br, 1H), 4.68 (br, 2H), 4.37 – 4.28 (m, 4H), 4.19 (s, 5H), 3.85 (s, 2H), 3.67 (s, 24H), 3.40 – 3.21 (m, 14H), 2.98 – 2.68 (m, 28H), 2.67 – 2.29 (m, 40H), 1.92 –1.82 (m, 2H), 1.64 –1.51 (m, 2H), 1.45 – 1.20 (m, 12H). IR (cm^{-1}): *υ* 1731.76 (-COOC-).

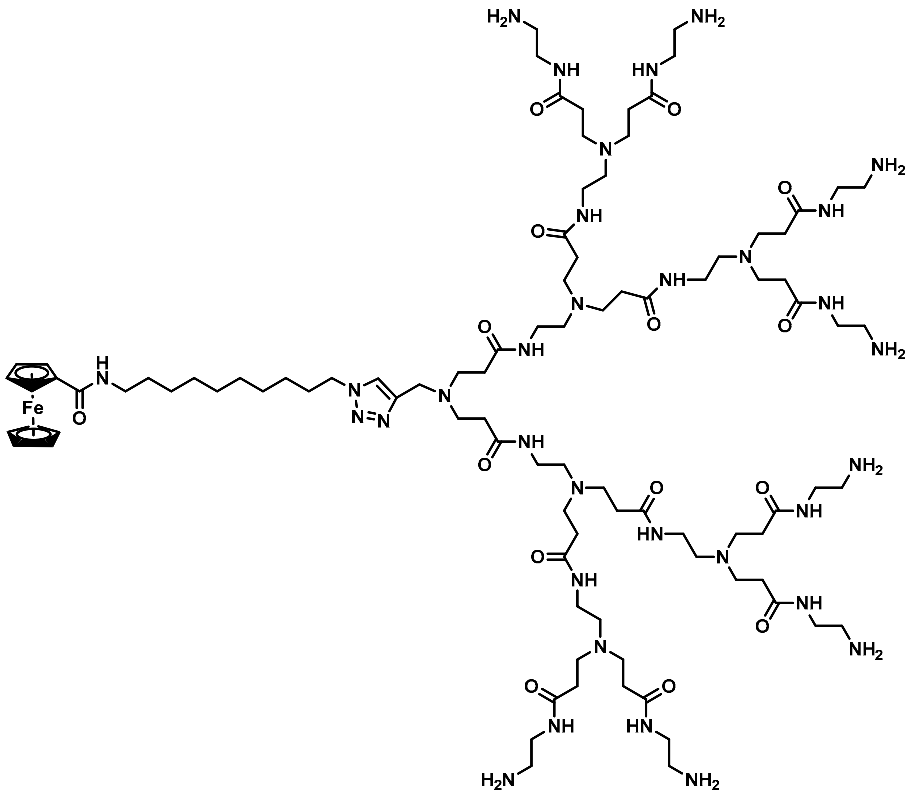

Fc-C$_{10}$-AmD 8A: **Fc-C$_{10}$-AmD 8E** (67.6 mg, 0.04 mmol) and methanol (1.8 mL) were taken into a reaction flask and protected by argon. And then ethylenediamine (1.8 mL, 27.0 mmol) was added dropwise with stirring at -20°C. After the addition of ethylenediamine, the reaction system was brought back to room temperature, and then the reaction was carried out at 30°C under dark. The rest of the synthesis was carried out according to **Fc-C$_6$-AmD 8A**, and **Fc-C$_{10}$-AmD 8A** (55.8 mg, 73%) was obtained as a yellow solid. ^{1}H NMR (300 MHz, $CD_3OD/CDCl_3$) δ 7.87 (s, 1H), 4.79 (br, 2H), 4.44 – 4.32 (m, 4H), 4.19 (s, 5H), 3.82 (s, 2H), 3.45 – 3.16 (m, 30H), 2.93 – 2.68 (m, 44H), 2.66 – 2.53(m, 12H), 2.50 – 2.16 (m, 28H), 1.97 – 1.85 (m, 2H), 1.66 – 1.53 (m, 2H), 1.46 – 1.23(m, 12H). ^{13}C NMR (75 MHz, $CD_3OD/CDCl_3$): δ 175.52, 174.94, 145.36, 125.29, 72.07, 71.26, 69.78, 54.08, 51.77, 43.41, 42.54, 42.54, 40.90, 39.13, 35.48, 35.25, 31.68, 31.21, 31.04, 31.04, 30.65, 30.43, 28.40, 27.71. ESI-HRMS(*m*/*z*): calcd for $C_{94}H_{175}FeN_{33}O_{15}$, $[M+2H]^{2+}$ 1032.6737, found 1032.6722. HPLC (RT = 20.2 min). IR (cm^{-1}): υ 1630.66 and 1542.24 (-NH(CO)-).
